# Early gestational mesenchymal stem cell secretome attenuates experimental bronchopulmonary dysplasia in part via exosome-associated factor TSG-6

**DOI:** 10.1186/s13287-018-0903-4

**Published:** 2018-06-26

**Authors:** Sushma Chaubey, Sam Thueson, Devasena Ponnalagu, Mohammad Afaque Alam, Ciprian P. Gheorghe, Zubair Aghai, Harpreet Singh, Vineet Bhandari

**Affiliations:** 1 Section of Neonatal-Perinatal Medicine, Department of Pediatrics, Drexel University College of Medicine, 245 N 15th Street, Philadelphia, PA 19102 USA; 2 Department of Pharmacology & Physiology, Drexel University College of Medicine, 245 N 15th Street, Philadelphia, PA 19102 USA; 3 Department of Obstetrics and Gynecology, Loma Linda University School of Medicine, 11370 Anderson Street, Loma Linda, CA 92354 USA; 4 Divison of Neonatology, Department of Pediatrics, Thomas Jefferson University Hospital, 132S, 10th Street, Philadelphia, PA 19107 USA; 5 Department of Medicine, Division of Cardiology, Drexel University College of Medicine, 245 N 15th Street, Philadelphia, PA 19102 USA

**Keywords:** Bronchopulmonary dysplasia, Mesenchymal stem cells, Wharton’s jelly, Secretome, Exosomes, Newborn, Pulmonary hypertension, Hyperoxia-induced lung injury, TSG-6

## Abstract

**Background:**

Mesenchymal stem cells (MSCs) are promising tools for the treatment of human lung disease and other pathologies relevant to newborn medicine. Recent studies have established MSC exosomes (EXO), as one of the main therapeutic vectors of MSCs in mouse models of multifactorial chronic lung disease of preterm infants, bronchopulmonary dysplasia (BPD). However, the mechanisms underlying MSC-EXO therapeutic action are not completely understood. Using a neonatal mouse model of human BPD, we evaluated the therapeutic efficiency of early gestational age (GA) human umbilical cord (hUC)-derived MSC EXO fraction and its exosomal factor, tumor necrosis factor alpha-stimulated gene-6 (TSG-6).

**Methods:**

Conditioned media (CM) and EXO fractions were isolated from 25 and 30 weeks GA hUC-MSC cultures grown in serum-free media (SFM) for 24 h. Newborn mice were exposed to hyperoxia (> 95% oxygen) and were given intraperitoneal injections of MSC-CM or MSC-CM EXO fractions at postnatal (PN) day 2 and PN4. They were then returned to room air until PN14 (in a mouse model of severe BPD). The treatment regime was followed with (rh)TSG-6, TSG-6-neutralizing antibody (NAb), TSG-6 (si)RNA-transfected MSC-CM EXO and their appropriate controls. Echocardiography was done at PN14 followed by harvesting of lung, heart and brain for assessment of pathology parameters.

**Results:**

Systemic administration of CM or EXO in the neonatal BPD mouse model resulted in robust improvement in lung, cardiac and brain pathology. Hyperoxia-exposed BPD mice exhibited pulmonary inflammation accompanied by alveolar-capillary leakage, increased chord length, and alveolar simplification, which was ameliorated by MSC CM/EXO treatment. Pulmonary hypertension and right ventricular hypertrophy was also corrected. Cell death in brain was decreased and the hypomyelination reversed. Importantly, we detected TSG-6, an immunomodulatory glycoprotein, in EXO. Administration of TSG-6 attenuated BPD and its associated pathologies, in lung, heart and brain. Knockdown of TSG-6 by NAb or by siRNA in EXO abrogated the therapeutic effects of EXO, suggesting TSG-6 as an important therapeutic molecule.

**Conclusions:**

Preterm hUC-derived MSC-CM EXO alleviates hyperoxia-induced BPD and its associated pathologies, in part, via exosomal factor TSG-6. The work indicates early systemic intervention with TSG-6 as a robust option for cell-free therapy, particularly for treating BPD.

**Electronic supplementary material:**

The online version of this article (10.1186/s13287-018-0903-4) contains supplementary material, which is available to authorized users.

## Background

Bronchopulmonary dysplasia (BPD) is a serious long-term complication of prematurity and the most common chronic lung disease in infants who need respiratory support after birth [1]. Brain injury and pulmonary hypertension (PH) are common complications of BPD resulting in increased morbidity and mortality [2–4]. There is no single effective therapy for BPD, making it important to explore new strategies of treatment. Mesenchymal stromal cell (MSC) therapy has been demonstrated to exert therapeutic effects in animal models of injury in the developing lung [5, 6]; however, a better understanding of the most appropriate cell type, optimal transplantation conditions and importantly, mechanism of action of MSC transplantation is required [7]. Beneficial effects of MSCs have been reported even in the absence of significant engraftment of donor cells in the recipient lungs following MSC therapy, suggesting that the therapeutic mechanism of action are mainly paracrine [5, 8, 9]. Several reports demonstrate better protection of structural deficits in preclinical BPD models using cell-free conditioned media (CM) of MSCs over MSCs themselves [5, 10–12]. Administration of MSC extracellular vesicles (EVs) has been reported to be beneficial in a number of lung disease models [9, 12–17] and ex vivo human lung models [15]. Willis et al group reported that MSC exosomes (EXO) act, at least in part, through modulation of lung macrophage phenotype, suppressing lung inflammation and immune responses to favor proper organ development [17]. The detailed mechanism of action or specific molecules involved in the action of MSC-CM EXO is yet to be reported.

Premature babies with extremely low birth weight most at risk for developing BPD are usually born between 24 weeks (wks) gestational age (GA) and 30 wks GA. Earlier studies demonstrate that higher levels of CD34+ hematopoietic stem and progenitor cells (HSPC) circulate in the blood of premature neonates, which may be associated with accelerated recovery from lung injury [18]. With the concept of using the human umbilical cord (hUC) MSCs from these premature babies for their therapy, we investigated the therapeutic efficiency of the early GA UC-MSCs secretome, both CM and EXO, on improvement in organ function and other markers of BPD pathology. We isolated MSCs from Wharton’s jelly (WJ) of hUC of premature neonates born at 25 wks and 30 wks GA to study whether the therapeutic effects are mediated through MSC-derived CM/secretome, specifically, EXO. MSC-CM and MSC-CM-derived EXO were administered to a mouse model of hyperoxia-induced injury that replicates human BPD [19–22]. We also identified MSC-secreted EXO factor tumor necrosis factor alpha-stimulated gene-6 (TSG-6), an immunomodulatory molecule, and evaluated its efficiency in treatment of BPD in our experimental mouse model. This strategy was used to explore the possibility of administering secreted factors as an alternative to the use of live cells.

In the present study, treatment of BPD mice with early GA UC-MSC-CM or EXO show robust decrease in lung inflammation, morphometric deformations, cell death, vascularization deficits, brain cell death and improved PH. Hypomyelination and decreased astrocytes in brain were reversed on intraperitoneal administration of MSC-CM EXO. We report presence of TSG-6 protein in the EXO fraction of 25 wks GA hUC MSC-CM. Intraperitoneal (i.p.) administration of TSG-6 improved BPD pathology in lungs, heart and brain, thus indicating the significance of its use in the systemic therapy of BPD. Administration of EXO isolated from TSG-6 small interfering ribonucleic acid (siRNA)-transfected MSCs abrogated the therapeutic effects of MSC-CM EXO in a BPD model. Additionally, we demonstrate similar decrease in EXO therapeutic effects in BPD mice on administration of TSG-6 neutralizing antibody. Our results demonstrate for the first time that TSG-6, found in the WJ MSC secretome, is one of the important therapeutic mediators for BPD and its associated pathologies, at least in part.

## Methods

### Animals

Wildtype (WT) C57BL/6 obtained from The Jackson Laboratory (Bar Harbor, ME, USA) were maintained in a breeding colony at Drexel University, Philadelphia, PA, USA. Animal procedures were performed in accordance with the NIH Guide for the Care and Use of Laboratory Animals and were approved by the Institutional Animal Care and Use Committee (IACUC) of Drexel University.

### Oxygen exposure and mouse model of BPD

Newborn (NB) pups were exposed to hyperoxia, along with their mothers, in cages in an airtight Plexiglas chamber (OxyCycler; Biospherix, Redfield, NY, USA) as described previously by our group [19, 20, 22–26]. Exposure to > 95% oxygen from birth or postnatal day 1 (PN1) until PN4, corresponds to the saccular stage of mouse lung development. At PN5, all animals were placed in room air (RA) up to PN14, which corresponds to the alveolar stage of mouse lung development, to allow for a period of recovery. Using this experimental model, NB WT mouse lungs at PN14 have the phenotype mimicking severe BPD in humans.

Isolation, expansion and characterization of hUC WJ MSCs. UCs were collected from healthy donors after their pre-term deliveries (25 and 30 wks GA) and immediately transferred to the laboratory. The collection of UC was approved by the Drexel University Institutional Review Board with a waiver of consent, as UCs are considered discarded material. MSCs were isolated from the WJ of hUC and expanded in MSC culture media, using a modified protocol [27] (Additional file 1: Experimental procedures). Characterization of UC-MSCs was performed as per the International Society of Cell Therapy (ISCT) guidelines. The UC-MSCs were characterized by standard fluorescence-activated cell sorting (FACS) for the expression of CD105, CD73 and CD90 and absence of HLA-DR, CD19 and CD14 surface molecules. UC-MSCs were differentiated for 21 days, into multilineage adipocytes, osteoblasts and chondroblasts, using specific differentiation media (StemPro Differentiation Kit, Gibco by Life Technologies, Carlsbad, CA, USA) and stained with Oil Red O, Alizarin S Red and Alcian Blue, respectively (Additional file 1: Experimental procedures).

### Preparation of UC MSC-CM, EXO and EXO-depleted CM for injections

To obtain MSC-CM, UC-MSCs at passage 3, with 70–80% confluency and cell equivalents of 0.76 × 10^6^ were washed three times with phosphate-buffered saline (PBS). The complete media of the cell cultures were replaced with serum-free [without fetal bovine serum (FBS)] DMEM:F12 containing 1% (v/v) penicillin/streptomycin. After 24 h, the serum-free media (SFM) were centrifuged (Eppendorf, Jericho, NY, USA) at 300 × g for 6 min at 4 °C, filtered through a 0.22 μm filter (Pall Corporation, Port Washington, NY, USA) and stored at −80 °C until use. For in vivo injections, the CM was concentrated tenfold using Amicon Ultra Centrifugal Filter Device (Millipore, Billerica, MA, USA) with a 10 kDa molecular weight cutoff. Total protein concentration of the secretome was measured by BCA kit (Pierce, Rockford, IL, USA) as per the manufacturer’s recommendations. A volume of 100 μl MSC-CM concentrate, equivalent to a total of 10 μg MSC-CM protein per mouse was injected via i.p. route at PN2 and PN4 to test the potential therapeutic effect of the treatment. The same amount of the concentrated DMEM:F12 media served as control injections.

Isolation of EXO from UC MSC-CM was performed using modified Thery’s protocol [28]. Briefly, the UC-MSC CM from passage 3 and cell equivalents of 0.76 × 10^6^ were centrifuged at 300 g followed by 2000 g for 10 min at 4 °C and filtered through a 0.22 μm filter. The filtrate was spun at 110,000 g for 2 h at 4 °C. The pellet was washed in PBS and spun at 110,000 g for 1.5 h at 4 °C. The obtained pellet of EXO was resuspended in PBS. 100 μl of EXO suspension, equivalent to a total of approximately 2.4 μg of MSC-CM EXO protein per mouse, and 4.5 × 10^8^ and 2.88 × 10^7^ particles (for MSC-CM EXO 25 wks and 30 wks, respectively), was injected via the i.p. route at PN2 and PN4. 100 μl of PBS served as control.

EXO-depleted CM 25 wks was obtained by centrifugation of 25 wks GA UC-MSC CM at 300 g, followed by 2000 g for 10 min at 4 °C. The supernatant was filtered through a 0.22 μm filter. The filtrate was spun at 110,000 g for 2 h at 4 °C. The EXO-depleted supernatant obtained after ultracentrifugation was carefully collected and concentrated tenfold using 10 kDa molecular weight cutoff Amicon Ultra Centrifugal Filter Device (Millipore, Billerica, MA, USA). Total protein concentration of the EXO-depleted CM was measured by BCA kit (Pierce, Rockford, IL, USA). A volume of 100 μl EXO-depleted MSC-CM concentrate was injected via the i.p. route at PN2 and PN4 to test the potential therapeutic effect of the treatment.

### Exosome dosing

Exosome preparations (100 μl of WJMSC-EXO) were injected i.p. at PN2 and PN4 in our BPD model, after diluting with PBS to achieve a standard dosage per pup corresponding to the product generated by 0.7 × 10^6^ MSCs over 24 h (h). There is no established quantification method for EXO-based therapeutics at present. Our rationale for choosing this EXO dose was based on other pilot experiments in the lab and previous studies by other groups, where they found that injecting mouse pups with a bolus dose of concentrated MSC-CM corresponding to the amount conditioned by 0.5 × 10^6^ to 1 × 10^6^ MSCs for 24 h − 36 h was sufficient to prevent lung injury and to reverse PH [10, 11, 29]. The particle count used in this study was 4.5 × 10^8^ particles for 25 wks EXO and 2.88 × 10^7^ particles for 30 wks EXO, protein concentration 2.8 μg and 2.4 μg, respectively for each, obtained from CM from MSCs grown in SFM, cell equivalent of 0.7 × 10^6^ for 24 h. Our dose is comparable to the recently published work by Willis et al [17], which injected a dose comprising of particle count of 8.5 × 10^8^ and protein concentration of 0.9 μg obtained from the MSC cell equivalent of 0.5 × 10^6^ cells for 36 h (Additional file 1: Table S1). Exosomal preparations for different GA MSCs was prepared maintaining identical conditions and handling to minimize the residual non-exosomal protein contamination and to maintain the consistency of the EXO fraction for both 25wks and 30 wks EXO for comparison.

### Bronchoalveolar lavage (BAL)

The mouse pups were euthanized for aspiration of BAL fluid (BALF) from the lungs. BALF total cell counts, absolute neutrophil count, macrophage percentage and total BALF protein was estimated as described in the Additional file 1: Experimental procedures.

### Tissue processing

The lung, heart and brain were excised after transcardiac perfusion with ice-cold PBS. The lungs were processed as previously described [19, 20, 22, 24]. The left lobes of lungs were subjected to standard protocol for lung inflation (25 cm) and fixed overnight in 4% paraformaldehyde (PFA) at 4 °C [19, 20, 22, 24]. The right lobes of the lungs were snap-frozen in liquid nitrogen and stored at −80 °C for biochemical analysis. Heart and brain were paraffin-embedded after overnight fixation in 4% PFA at 4 °C. Before embedding, the brain was divided into four regions - olfactory bulb, forebrain, mid brain and hind brain for analysis.

### Lung morphometry

Five-micrometer-thick paraffin-embedded sections were stained with hematoxylin and eosin (H&E) as previously described [30]. A minimum of five randomly chosen areas from each section were photographed with the ×200 magnification. Investigators were blinded to experimental groups for the analysis. Alveolar size was estimated from the mean chord length of the airspace, as described previously [22, 26]. Alveolar septal wall thickness was estimated using ImageJ software, adapting the method for bone trabecular thickness, for the lung [26, 31, 32]. The mean alveolar area was calculated using the method previously reported [33]. Number of branches, junctions, junction voxels, triple points and quadruple points were calculated using AnalyzeSkeleton program from ImageJ. This plugin tags all pixel/voxels in a skeleton image and then counts all its junctions, triple and quadruple points and branches. Junction voxels are defined by having more than two neighbors. Number of triple points and quadruple points depicts those cells having more than three or four neighbors, respectively.

### Western blot analysis, dot blot assay, terminal deoxynucleotidyl transferase dUTP nick-end labeling (TUNEL) assay, immunofluorescence and immunohistochemistry and enzyme-linked immunosorbant assay (ELISA) for interleukin-6 (IL-6) in lung lysate

Described in Additional file 1: Experimental procedures.

### Heart measurements for PH-induced right ventricular hypertrophy (RVH)

Cross-section of paraffin-embedded heart from four to six mice from each group were analyzed for measurement of right ventricular (RV) to left ventricular (LV) diameter ratio, [RV/LV] and RV to LV + interventricular septa (IVS) ratio, [RV/(LV + IVS)], also called Fulton’s index to quantitate the degree of PH-induced RVH, as previously described [19, 26].

### Echocardiography

Mouse pups at PN14 were anesthetized using i.p. ketamine/xylaxine injections (100/10 mg/kg mouse weight). Echocardiography was performed in anesthetized mice using the Vevo 2100 imaging system (Visual Sonics, Toronto, Canada) with a high frequency (18–38 MHz) probe (VisualSonics MS400) with simultaneous ECG recording. Visual sonic software analysis tool was used to obtain pulmonary artery acceleration time (PAAT) and PA ejection time (PAET) values. A short PAAT or small PAAT/PAET ratio indicates high peak systolic PA pressure, as previously described and validated [34, 35].

### Transmission electron microscopy (TEM)

For EXO visualization and morphological assessment, an aliquot from an EXO preparation (3–5 μl) was adsorbed for 15 s to a formvar-carbon coated grid (Electron Microscopy Sciences, Hatfield, PA, USA). Excess liquid was removed with Whatman Grade 1 filter paper (Sigma-Aldrich, St. Louis, MO, USA) followed by staining for 15 s with 2% uranyl acetate. Adsorbed exosomes were examined on a JEOL 1010 transmission electron microscope (TEM), and images were recorded with a Hamamatsu digital camera using a magnification of ×100,000 (Hamamatsu, Photonics, Hamamatsu City, Japan).

### Nanoparticle tracking analysis (NTA)

Size and concentration distributions of exosomes were determined using nanoparticle tracking analysis (NS-300 NanoSight Instrument, Malvern Instruments Ltd., Malvern, UK). NTA determines the Brownian motion of nanoparticles in real-time to assess size and concentration utilizing a laser-illuminated microscopic technique equipped with a 405 nm laser and a high sensitivity digital camera system (sCMOS camera, Hamamatsu Photonics, Hamamatsu, Japan).

EXO samples were diluted in vesicle-free PBS. Samples were administered and recorded under controlled flow, using the NanoSight syringe pump. Data acquisition and processing were performed using NTA software version 2.3 build 0025. Background extraction was applied, and automatic settings were employed to determine the minimum expected particle size, minimum track length, and blur settings. Since samples were diluted in ultrapure DPBS 0.0095 M (PO_4_) w/o Ca and Mg (Lonza, Basel, Switzerland), viscosity settings for water were applied and automatically corrected for the temperature used. Data were obtained at camera-level 12 (shutter:600, gain: 350). For each sample, three videos of 30 s duration at 25 frames per second were recorded and assigned a single measurement in triplicates. Three sets of samples were run, from which exosome distribution, size and mean concentration were calculated.

### RNA isolation and quantitative real-time PCR (qRT-PCR) of human lung tracheal aspirates and mouse lung tissues

Collection and processing of the human lung samples was approved by the institutional review board of Thomas Jefferson University Hospital. Human lung tracheal aspirates (TA) were obtained from premature infants being mechanically ventilated in the first PN week with an in-dwelling endotracheal tube. These infants had the final outcomes of having the diagnoses of with or without BPD. Selected clinical details are shown in Additional file 1: Table S2.

Pellets obtained from TA were subjected for total RNA extraction using TRIZOL (Invitrogen, Carlsbad, CA, USA) and RNAeasy kit (Qiagen, Hilden, Germany). First-strand cDNA was synthesized with iScript cDNA Synthesis kit for Real-Time-PCR (Bio-Rad, Hercules, CA, USA) according to the manufacturer’s instructions. Real-time PCR reaction was performed in a 20 μL volume with SYBR Green (Bio-Rad, Hercules, CA, USA) with the use of pooled cDNA samples. Human TSG-6 primers (Applied Biosystems, Foster City, CA, USA) used for the amplification: forward primer: ACTCAAGTATGGTCAGCGTATTC (sense) and reverse primer: GCCATGGACATCATCGTAACT (antisense). human HPRT, forward primer 5′-TAT GGC GAC CCG CAG CCC T-3’ reverse primer 5’-CAT CTC GAG CAA GAC GTT CAG-3′.

RNA was isolated from excised mouse lung tissues from RA, BPD and BPD groups injected with PBS, MSC-CM EXO 25 wks, TSG-6 using TRIZOL (Invitrogen, Carlsbad, CA, USA) and RNAeasy kit (Qiagen, Hilden, Germany). For gene expression analysis of IL-6, tumor necrosis factor alpha (TNF-α) and interleukin 1 beta (IL-1β), real-time PCR was performed using the following primers: IL-6: forward 5′-TGG GGC TCT TCA AAA GCT CC-3′, reverse 5′-AGG AAC TAT CAC CGG ATC TTC AA-3′; TNF-α: forward 5′- GGG TCG CAC CAT GAA GGA G-3, reverse: 5′- GAA GTG GTA GTG GTA GCT TTC G-3′; IL-1β: forward 5’ GCA CTA CAG GCT CCG AGA TGA AC-3′, reverse: 5′-TTG TCG TTG CTT GGT TCT CCT TGT-3’ HPRT: forward: 5’-GCT GGT GAA AAG GAC CTC T-3′, reverse: 5’-CAC AGG ACT AGA ACA CCT GC-3′. The reaction was performed at 95 °C for 10 min followed by a 40-cycle denaturation at 94 °C for 15 s, annealing at 54 °C for 30 s and extension at 72 °C for 40 s, using a Real-Time System (Applied Biosystems, Foster City, CA, USA).

### Transfection of hMSCs with TSG-6 siRNA

hUC MSCs from passage 3 were thawed and plated at 200 cells/cm^2^ in multiple six-well plates in DMEM:F12 media with antibiotics. The culture medium was changed every 2 days. After incubation for 4–5 days, when the cells were ~ 80% confluent, the cells were incubated in SFM for 12 h followed by transfection with TSG-6 siRNA (sc-39,819; Santa Cruz Biotechnology, Dallas, TX, USA) or negative control [scrambled siRNA (scr siRNA)/control siRNA, sc-37,007; Santa Cruz Biotechnology, Dallas, TX, USA] using a commercial kit (Lipofectamine 3000 reagent; Invitrogen, Carlsbad,, CA, USA) as per the manufacturer’s instructions. A 5 μM stock solution of TSG-6 siRNA or negative control (scr siRNA/control siRNA) was diluted with reagent (Lipofectamine 3000 Reagent; Invitrogen, Carlsbad,, CA, USA), which was further diluted with transfection medium (siRNA Transfection medium, Santa Cruz Biotechnology, Dallas, TX, USA). The mixture was incubated for 30 min at room temperature. The mixture, together with transfection medium, was added to the cells. Sixteen hours later, the transfection medium was replaced with DMEM:F12 media containing 10% FBS. hMSCs were revived for 6 h. The culture was then incubated in complete media with antibiotics for another 16 to 20 h. For EXO isolation from the TSG-6 siRNA-transfected MSCs, TSG-6 siRNA transfected MSCs were grown in SFM for another 24 h. After 24 h, serum-free CM was collected for isolation of TSG-6 siRNA EXO. To confirm knockdown of TSG-6, RNA was extracted from scrambled (scr) and TSG-6 siRNA-transfected MSCs (RNeasy Mini Kit; Qiagen, Hilden, Germany) and assayed for TSG-6 by real-time RT-PCR using TSG-6 primers.

### Knockdown of TSG-6 in MSC-CM EXO-injected BPD mice using TSG-6 neutralizing antibody (NAb)

Newborn pups were exposed to hyperoxia (> 95% oxygen) from birth to PN4. At PN5, all animals were placed in room air (RA) up to PN14, to allow for a period of recovery. To analyze if NAb against TSG-6 affects the therapeutic properties of EXO, we administered TSG-6 NAb (A38.1.20, Santa Cruz Biotechnology, Dallas, TX, USA, 5 μg/dose) intraperitoneally, 1 day before EXO 25 wks injections (i.e., at PN1 and PN3) and 1 day after the second injection (i.e., at PN5). Isotype IgG (R&D Systems, Minneapolis, MN, USA, 5 μg/dose) was used as control injections for the NAb groups and were administered at PN 1, 3 and 5. The pups were kept at RA till PN14 for echo analysis and harvesting the tissues for other analysis.

### Statistical analysis

Statistical analyses were performed using one-way analysis of variance (ANOVA) followed by Tukey’s post hoc test for comparison between three or more groups using GraphPad Prism software 7.0 for Windows (GraphPad Software, San Diego, CA, USA). Statistical significance was defined as *p* < 0.05. Mean values were expressed as mean ± SEM. The number of mice/group is indicated in the legend of each figure.

## Results

### Isolated preterm hUCs MSC, but not fibroblast, injections improve the BPD pulmonary phenotype

In initial experiments, we isolated MSCs from preterm (32 weeks) hUCs and injected them in our mouse BPD model. Human primary dermal fibroblast (HDF) cells obtained from American Type Culture Collection (ATCC) (Manassas, VA, USA) were also injected, as control cells, in the BPD mice. HDF were grown as per instructions provided with the cells. As shown in Additional file 1: Figure S1, chord length, indicative of alveolar size, was increased in the BPD mice, with no difference as compared to the BPD mice injected with fibroblasts. On the other hand, hUC MSC-injected BPD mice lungs showed an improvement in the pulmonary phenotype, as evidenced by significant decrease in chord length value compared to the other two BPD groups (Additional file 1: Figure S1).

### Isolation of hUC MSCs and collection of UC-MSC CM and EXO for injection into BPD mouse model

hUC MSCs were isolated and characterization of UC-MSCs was performed as per the International Society of Cell Therapy (ISCT) guidelines. The isolated WJ-MSCs were adherent to plastic, displayed a fibroblast-like phenotype and showed unaltered viability (Additional file 1: Figure S2A). Characteristic cell surface markers of MSCs were identified at passage 3 by flow cytometry. Cells were positive for CD105, CD73 and CD90 and prominently negative for hematopoietic stem cell markers HLA-DR, CD19 and CD14 surface molecules as shown by standard FACS (Additional file 1: Figure S2B). UC-MSCs were expanded in culture up to passage 5 to study their growth kinetics. There was no significant difference between mean population doubling time for 25 and 30 wks GA UC MSCs, ~ 33.5 h and ~ 35 h, respectively. On differentiation, UC-MSCs differentiated into multilineage adipocytes, osteocytes and chondrocytes as detected by staining with Oil Red O, Alizarin S Red and Alcian Blue, respectively (Additional file 1: Experimental procedures and Figure S2C).

To study whether the secretome of MSCs plays an important role in its therapeutic effects, we used CM and EXO from UC MSCs as a feasible i.p. treatment in our well-established murine hyperoxia-induced BPD model [19, 20, 24, 26]. CM was collected from MSCs incubated with SFM for 24 h at 37 °C in the CO_2_ incubator. Culturing MSCs in SFM after washing the monolayer with PBS allowed us to ensure that no exosomal contamination from the FBS is carried over to the MSC-CM collected, and all EXO present in the CM, after 24 h incubation, are those secreted from the MSCs. MSC-CM was concentrated tenfold and was injected intraperitoneally into the BPD mouse model.

### Isolation of EXO from the CM and its characterization

EXO were isolated from the CM by serial centrifugation following modified Thery’s protocol [28] as described in the Additional file 1: Experimental procedures. Isolated EXO were characterized by electron microscopy (EM) for morphology and size (Additional file 1: Figure S3A). EM revealed a heterogeneous exosome population of MSC-CM EXO, having a typical diameter of 40–140 nm. Particle number administered in each dose as analyzed by NTA particle number: 25 wks EXO 4.5 × 10^8^ particles, 30 wks EXO 2.88 × 10^7^ particles. Representative plots of the 25 wks EXO and 30 wks EXO are shown in Additional file 1: Figure S3B. The 25 wks EXO and 30 wks EXO samples were diluted 1:50 and 1:20, respectively, for the analysis. Dot blot was performed, loading equal amounts of protein, to characterize isolated EXO after probing with exosome-specific surface marker, CD63. Trans-Golgi network protein (TGN48) was used as a negative control for the EXO. CD63 was expressed in the CM and EXO fractions of 25 and 30 wks CM, demonstrating the presence of EXO in both CM and EXO fractions (Additional file 1: Figure S3C). No signal for TGN48 was detected in the EXO fraction of both 25 and 30 wks CM, though the signal was seen in the CM depicting that the EXO fraction isolated is pure and does not have cytoplasmic membranes (Additional file 1: Figure S3D). No signal was detected for DMEM:F12 and PBS fractions, which are devoid of any cells and cellular compartments. Western blotting detected characteristic exosomal marker CD81 (25 kDa) in the EXO fraction of MSC-CM, both at 25 wks and 30 wks **(**Additional file 1: Figure S3E). Specific signal for the exosomal marker Alix-1 (97 kDa) was also detected for 25 wks GA MSCs, CM and EXO samples by Western blotting (Additional file 1: Figure S3F). I.p. injections of MSC-CM EXO from 25 and 30 wks GA UC were done after protein concentration determination, as described in the Methods, to determine the effect of EXO in the BPD mouse model.

### MSC secretome treatment reverses hyperoxia-induced pulmonary inflammation and alveolar-capillary leak in the BPD mouse model

**Fig. 1:**
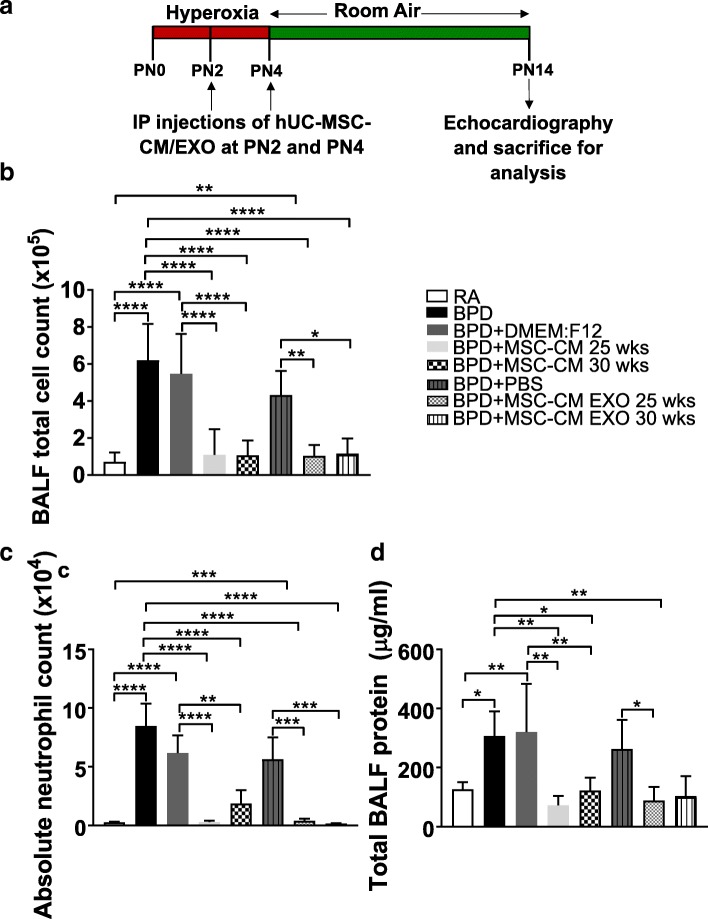
hUC MSC secretome treatment reverses pulmonary inflammation and alveolar-capillary leak associated with hyperoxia-induced lung injury in the BPD mouse model. **a** Schematic representation of BPD mouse model and secretome injection regime. The mice were kept in 100% oxygen from birth to PN4 followed by RA exposure till PN14. The mice were used for echocardiography and sacrificed for analysis at PN14. IP injections of hUC MSC-CM or hUC MSC-CM EXO were given at PN2 and PN4. 10 μg of MSC-CM and 2.5 μg of MSC-CM EXO (GA 25 wks and 30 wks), resuspended in 100 μl of PBS respectively, was injected into each neonatal mouse at PN2 and PN4. **b-d** Histogram showing BALF total cell count (**b**), BALF absolute neutrophil count (**c**), total BALF protein (**d**), in RA, BPD and vehicle (DMEM:F12 or PBS)-injected, MSC-CM or EXO 25 wks-injected, MSC-CM or EXO 30 wks-injected BPD mice at PN14. All values are expressed as mean ± standard error of the mean (SEM); eight experiments, *N* = 3–9 mice per group; one-way ANOVA with Tukey’s post hoc correction; *p < 0.05; **p < 0.01; ***p < 0.001; ****p < 0.0001. *BALF* bronchoalveolar lavage fluid, *BPD* bronchopulmonary dysplasia, *CM* conditioned medium, *EXO* exosomes, *hUC* human umbilical cord, *i.p.* intraperitoneal, *MSC* mesenchymal stem cell, *PBS* phosphate-buffered saline, *PN* postnatal, *RA* room air

To determine if hyperoxia-induced lung inflammation responds to MSC paracrine signals, we performed i.p. injections of concentrated UC MSC-CM or UC MSC-CM EXO into pups (at PN2 and PN4) exposed to hyperoxia (> 95% O_2_). After 4 days hyperoxia exposure from birth to PN4, these pups were placed at RA until PN14, as defined in our hyperoxia-induced BPD mouse model (Fig. 1a) [19, 20, 24, 26]. The control group comprised of pups exposed to the same hyperoxia conditions and injected with vehicle (serum-free culture media-DMEM: F12 or PBS) at PN2 and PN4. Hyperoxia resulted in accumulation of inflammatory cells on injury, indicated by a statistically significant increase in total cell count in BALF in BPD compared to RA (Fig. 1b) mice. However, on MSC-CM or EXO treatment, BALF total cell count was statistically decreased to RA levels (Fig. 1b). No significant decrease in BALF total cell count was observed in BPD mice injected with the vehicle (DMEM:F12 or PBS). Similarly, there was a significant increase in infiltration of neutrophils, as assessed by the absolute neutrophil count, in BALF of BPD compared to RA. This increase in neutrophil count in BPD mice was blocked on treatment with MSC-CM or EXO 25 and 30 wks (Fig. 1c). No changes were noted in the percentages of BALF macrophages (Additional file 1: Figure S4A).

Hyperoxia-induced lung injury is characterized by endothelial cell damage and disruption of the alveolar-capillary barrier, leading to increased protein leak in the BALF. To determine the extent of capillary leakage, the protein concentration in BALF was measured (Fig. 1d). There was a statistically significant increase in BALF protein in BPD versus RA mice. This increase in total BALF protein in BPD was not diminished on treatment with DMEM:F12 or PBS; however, MSC-CM or EXO treatment significantly decreased the protein leak. In summary, our results show that MSC-CM and EXO treatment significantly suppressed inflammatory cell accumulation in the lung and has a protective role in the maintenance of the alveolar-capillary barrier in the presence of hyperoxia.

### MSC-CM or EXO treatment reverses alveolar injury, septal thickness and other morphometric alterations associated with hyperoxia-induced lung injury in the BPD mouse model

**Fig. 2:**
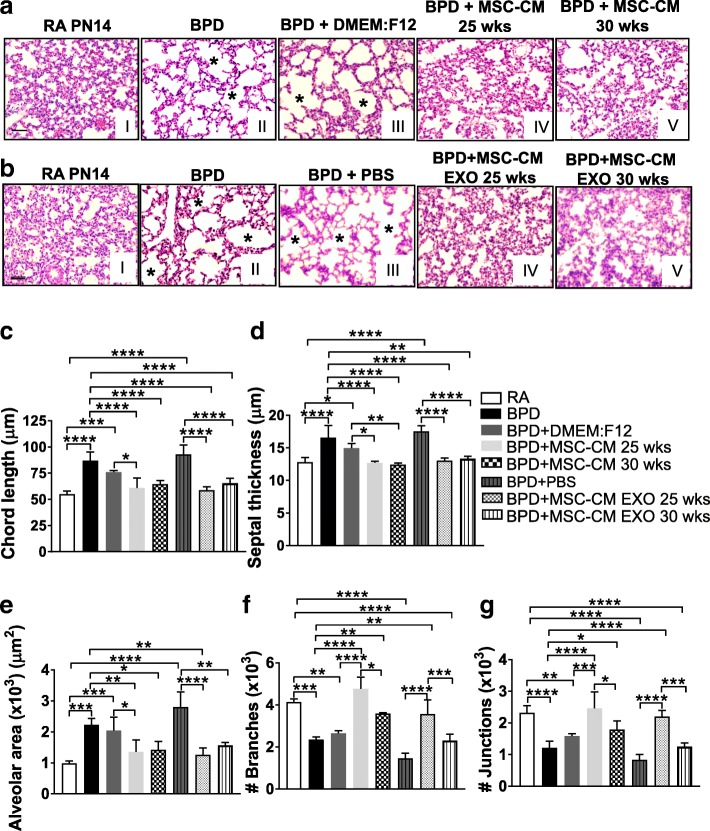
hUC MSC secretome treatment reverses altered lung morphology associated with hyperoxia-induced lung injury in the BPD mouse model. **a** Representative images of lung histology with H&E stain from the five experimental groups, RA (I), BPD (II), BPD + DMEM:F12 (III), BPD + MSC-CM 25 wks (IV), BPD + MSC-CM 30 wks (V). *Asterisks* depicts the increased alveolar simplification in the BPD and DMEM:F12-injected BPD mice as compared to RA. ×200 magnification, Scale bar: 50 μm. **b** Representative images of lung histology with H&E stain from the five experimental groups, RA (I), BPD (II), BPD + PBS (III), BPD + MSC-CM EXO 25 wks (IV), BPD + MSC-CM EXO 30 wks (V). *Asterisks* depict the increased alveolar simplification in the BPD and PBS-injected BPD mice as compared to RA. ×200 magnification, Scale bar: 50 μm. **c-g** Histogram depicting the mean chord length (**c**), septal thickness (**d**), alveolar area (**e**), number of branches (**f**), number of junctions (**g**) in lungs of RA, BPD, DMEM:F12 or PBS-injected, MSC-CM or EXO 25 wks-injected, MSC-CM or EXO 30 wks-injected BPD mice at PN14. All values are expressed as mean ± standard error of the mean (SEM); eight experiments, N = 3–7 mice per group; one-way ANOVA with Tukey’s post hoc correction; *p < 0.05; **p < 0.01; ***p < 0.001; ****p < 0.0001. *BPD* bronchopulmonary dysplasia, *CM* conditioned medium, *EXO* exosomes, *MSC* mesenchymal stem cell, *PBS* phosphate-buffered saline, *PN* postnatal, *RA* room air

Impaired alveolar growth, as evidenced by fewer and larger alveoli with heterogeneous sizes, was observed in BPD compared to RA lungs. These impairments in alveolar growth and morphological changes observed in BPD were attenuated in the MSC-CM or EXO-injected pups but not in DMEM:F12 or PBS-injected pups (Fig. 2a, b). Based on morphometric analysis, the chord length, which is indicative of alveolar size, was significantly higher in BPD as compared to RA groups. This hyperoxia-induced increase in mean chord length was significantly ameliorated by UC-MSC-CM or EXO treatment (Fig. 2c).

There was a statistically significant increase in alveolar septal thickness in BPD and DMEM:F12 or PBS-injected group compared to RA (Fig. 2d). This increase in septal thickness was significantly reduced to RA levels on administration of MSC-CM or EXO, both in 25 and 30 wks groups, depicting the therapeutic effect of the secretome (Fig. 2d). Alveolar area was significantly increased in BPD compared to RA lungs. Injecting the BPD mice with vehicle DMEM:F12 or PBS had no effect. However, alveolar area was significantly reduced to the RA levels after MSC-CM or EXO injections in BPD mice (Fig. 2e). Further in-depth analysis of other lung morphological parameters, such as number of branches, junctions (Fig. 2f, g), triple points and quadruple points (Additional file 1: Figure S4B-C) was performed. Interestingly, we found that although both 25 and 30 wks CM treatment attenuated the morphological alterations in BPD mouse model, CM or EXO treatment from earlier gestational age, 25 wks GA UC showed statistically significant improvement in selective lung morphometric parameters when compared to CM or EXO from 30 wks GA UC (Fig. 2f, g, Additional file 1: Figure S4B-C). To summarize, MSC-CM treatment significantly improved pulmonary architecture in the hyperoxia-induced mouse BPD model, with a preferential enhanced response from the CM or EXO derived from the 25 wks GA UC.

To further assess the mechanism of the improved architecture in lung tissue, we evaluated apoptosis using TUNEL assay. Hyperoxia causes oxidant-induced DNA injury and cell death that manifests as enhanced pulmonary tissue TUNEL staining. Apoptotic cells were seen significantly more in BPD compared to RA (Additional file 1: Figure S4D). MSC-CM or EXO treatment significantly decreased hyperoxia-induced cell death in the lungs of the BPD mice (Additional file 1: Figures S4D-E and S5A-B). Similar decrease in cell death on treatment with MSC-CM EXO was shown by cleaved caspase-3 staining (Additional file 1: Figure S5C-D). We further demonstrate that MSC-CM EXO treatment rescued hyperoxia-induced loss of peripheral pulmonary blood vessels in the BPD mice **(**Additional file 1: Figure S6). The immunofluorescence intensity of CD31 staining, a known marker of blood vessels, was significantly reduced in BPD as compared to RA in PN14 pups. This hyperoxia-induced loss of CD31 staining was significantly increased in the MSC-CM EXO-injected groups (Additional file 1: Figure S6).

### MSC secretome treatment reverses PH-induced RVH in the BPD mouse model

**Fig. 3:**
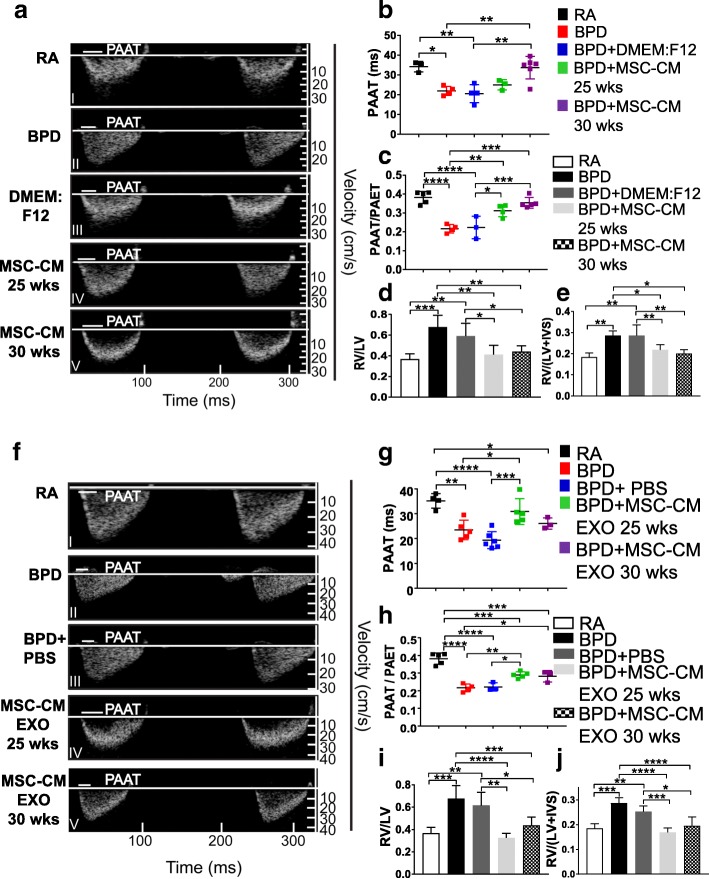
hUC MSC secretome treatment reverses PH and RV hypertrophy in hyperoxia-induced lung injury. **a** Echocardiography shows reversal of cardiac dysfunction on MSC-CM treatment in the BPD mouse model. The *white line* in the echocardiogram depicts PAAT values for the specific group, where PAAT is the pulmonary artery acceleration time. **b** Graph demonstrating PAAT values from the five experimental groups at PN14. **c** Graph depicting PAAT/PAET ratio where PAET is the pulmonary artery ejection time. **d** Histogram showing the RV to LV ratio in the five experimental groups. **e** Fulton’s index [RV/(LV + IVS)], reflecting right ventricular hypertrophy, in the five experimental groups at PN14. **f** Echocardiography shows reversal of cardiac dysfunction on MSC-CM EXO treatment in the BPD mouse model. The *white line* in the echocardiogram depicts PAAT values for the specific group. **g** Graph demonstrating PAAT values from the five experimental groups at PN14. **h** Graph depicting PAAT/PAET ratio where PAET is the pulmonary artery ejection time. **i** Histogram showing the RV to LV ratio in the five experimental groups. **j** Fulton’s index [RV/(LV + IVS)], reflecting right ventricular hypertrophy, in the five experimental groups at PN14. All values are expressed as mean ± standard error of the mean (SEM); 5 experiments, *N* = 3–7 mice per group; one-way ANOVA with Tukey’s post hoc correction; *p < 0.05; **p < 0.01; ***p < 0.001; ****p < 0.0001. *BPD* bronchopulmonary dysplasia, *CM* conditioned medium, *EXO* exosomes, *IVS* interventricular septa, *LV* left ventricular, *MSC* mesenchymal stem cell, *PAAT* pulmonary artery acceleration time, *PAET* pulmonary artery ejection time, *PBS* phosphate-buffered saline, *RA* room air, *RV* right ventricular

PAAT values from different treatment conditions were obtained by high-resolution echocardiography, as described before [35]. PAAT, as a surrogate of mean PA pressure, was found to be shortened in BPD animals and vehicle-injected (DMEM:F12) BPD mice **(**Fig. 3aII-III) but reverted to RA levels in MSC-CM 30 wks-treated BPD mice (Fig. 3a I, V, b). The ratio between PAAT and PAET show significant decrease in BPD and DMEM:F12-injected groups as compared to RA. However, on treatment with CM, there was significant increase in PAAT/PAET ratio, indicative of reversal of PH (Fig. 3c).

RVH, as depicted by RV to LV ratio, was statistically significantly increased in BPD when compared to RA. RVH was attenuated on MSC-CM treatment (Fig. 3d). Fulton’s index, calculated as [RV/(LV + IVS)], also determines RVH, was significantly increased in BPD pups as compared to RA pups (Fig. 3e). However, injections with MSC-CM significantly decreased the Fulton’s index to RA levels (Fig. 3e). MSC-CM EXO 25 wks treatment significantly increased the PAAT values of BPD mice to RA levels (Fig. 3f, g). Treatment with EXO from both 25 wks and 30 wks UC MSCs significantly increased PAAT/PAET ratio, indicative of reversal of PH. There was no difference between the BPD and PBS-injected groups depicting specific attenuation after MSC-CM EXO injections (Fig. 3h). We demonstrate that MSC-CM EXO treatment attenuated RVH and PH, as depicted by significantly decreased RV/LV ratio (Fig. 3i), and the Fulton’s index (Fig. 3j) in BPD mice.

### MSC secretome treatment show decreased hyperoxia-induced cell death in the brain in the BPD mouse model

To evaluate apoptosis levels in brain tissue, the mouse brain was divided into four different regions: olfactory bulbs, forebrain (comprising of corpus callosum and lateral ventricles), midbrain (comprising of corpus callosum and hippocampus) and hindbrain (comprising of cerebellum). TUNEL staining was performed in different regions of the brain. No difference in cell death between the RA, BPD, vehicle-injected (DMEM:F12 or PBS) and MSC-CM or EXO-injected groups was detected in the olfactory bulb region (Additional file 1: Figure S7A and E). Apoptotic cells were significantly more in BPD compared to RA in forebrain (Additional file 1: Figure S7B and F). MSC-CM treatment significantly reduced forebrain cell apoptosis (Additional file 1: Figure S7B). Statistically significant decrease in cell death was also seen in midbrain and hindbrain regions of MSC-CM or EXO-treated pups (Additional file 1: Figures S7 C-D and G-H), demonstrating that UC-MSC CM or EXO differentially attenuate cell death in brain, in different regions of the brain. No recovery was observed in the BPD pups injected with the vehicle injections (DMEM:F12 or PBS).

### MSC CM EXO reverses hypomyelination and glial fibrillary acidic protein (GFAP) expression in the brain of BPD mice

Myelin binding protein (MBP) is involved in development of white matter in the brain and is a marker of mature oligodendrocytes. We assessed brain myelination by immunofluorescent staining of corpus callosum region of RA, BPD and MSC-CM EXO-treated BPD mice for MBP at PN14 (Additional file 1: Figure S8A and C). BPD mice injected with PBS were used as controls. MBP mean pixel intensity, indicative of the extent of brain myelination, was significantly reduced in BPD and PBS-injected group compared to RA group (Additional file 1: Figure S8A I-III). However, on MSC-CM EXO treatment, MBP expression was significantly increased in BPD mice, thus depicting reduced loss of myelination in the brains of BPD mice (Additional file 1: Figure S8A IV-V and C). We evaluated the effect of MSC-CM EXO treatment on GFAP, a characteristic marker of astrocytes, levels in hippocampus of BPD brain at PN14. Immunofluorescent staining of the brain sections with GFAP show statistically significantly decreased mean pixel intensity in BPD compared to RA (Additional file 1: Figure S8 B I-II and D). However, on MSC-CM EXO treatment, mean pixel intensity of GFAP in BPD mice was significantly increased, suggesting an increase in astrocytes in the brains of treated BPD mice (Additional file 1: Figure S8 B IV, V and D).

### Therapeutic factor of UC-MSC secretome resides in the EXO fraction

**Fig. 4:**
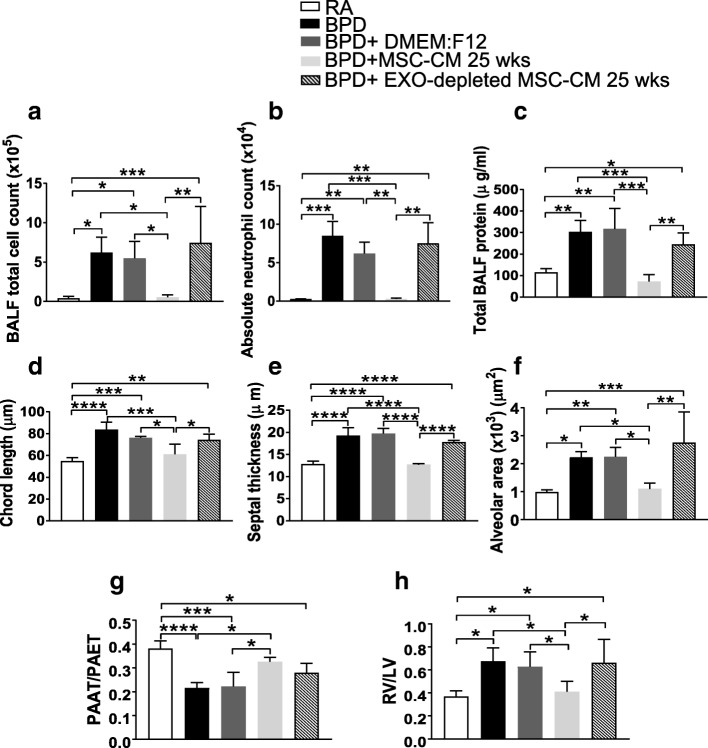
EXO-depleted MSC-CM 25 wks injections does not reverse hyperoxia-induced pulmonary inflammation, altered morphology, PH and RVH in the BPD mouse brain. **a-c** Histograms showing BALF total cell count (**a**), BALF absolute neutrophil count (**b**), total BALF protein (**c**) in RA, BPD, DMEM:F12-injected, MSC-CM 25 wks and EXO-depleted MSC-CM 25 wks-injected BPD mice at PN14. **d-f** Histograms showing the mean chord length (**d**), septal thickness (**e**), alveolar area (**f**) in lungs of RA, BPD, DMEM:F12-injected, MSC-CM 25 wks and EXO-depleted MSC-CM 25 wks-injected BPD mice. **g-h** Graph demonstrating PAAT/PAET ratio (**g**), RV to LV ratio (**h**), reflecting RV hypertrophy**,** in the five experimental groups at PN14. MSC-CM 25 wks data set used earlier in this manuscript was used for comparison with the EXO- depleted MSC-CM 25 wks group. All values are expressed as mean ± SEM; five experiments, N = 3–9 mice per group; one-way ANOVA with Tukey’s post hoc correction; *p < 0.05; **p < 0.01; ***p < 0.001; ****p < 0.0001. *BPD* bronchopulmonary dysplasia, *CM* conditioned medium, *EXO* exosomes, *LV* left ventricular, *MSC* mesenchymal stem cell, *PAAT* pulmonary artery acceleration time, *PAET* pulmonary artery ejection time, *PBS* phosphate-buffered saline, *RA* room air, *RV* right ventricular

MSC-CM comprises secretome of the MSCs, which includes EXO. Both MSC-CM and MSC-CM EXO fractions show improvement in BPD pathology on i.p. injections in the neonatal BPD mouse model. To determine if EXO is the therapeutic fraction of the secretome, we performed i.p. injections of concentrated EXO-depleted MSC-CM 25 wks into pups exposed to > 95% O_2_ in our hyperoxia-induced BPD mouse model (Fig. 4). The control group comprised of BPD pups injected with vehicle (serum-free culture media-DMEM:F12). We chose the 25 wks GA MSC-CM samples to deplete the EXO as the recovery was augmented with this early GA. To obtain EXO-depleted fraction, we utilized the CM supernatant obtained after pelleting down MSC-CM EXO 25 wks, during EXO isolation by ultracentrifugation. The EXO-depleted MSC-CM 25 wks obtained was tenfold concentrated to prepare for the injection regime (Fig. 4).

EXO-depleted MSC-CM 25 wks treatment had no effect on the hyperoxia-induced pulmonary inflammation and alveolar-capillary leak in the BPD mouse model. BALF total cell count (Fig. 4a), BALF absolute neutrophil counts (Fig. 4b) and total BALF protein concentration (Fig. 4c) were significantly high in EXO-depleted MSC-CM 25 wks-injected groups as compared to RA. EXO-depleted MSC-CM 25 wks-injected group showed no reversal in the morphometric alterations in the hyperoxia-induced lung injury (Fig. 4d-f and Additional file 1: Figure S9A-D). The mean chord length, septal thickness and alveolar areas were significantly higher in EXO-depleted MSC-CM 25 wks-injected groups compared to RA (Fig. 4d-f). Other lung morphometric parameters such as number of branches, junctions, triple points, quadruple points showed significant decrease in EXO-depleted MSC-CM 25 wks fraction, similar to that seen in the BPD and DMEM:F12-injected groups, when compared to RA (Additional file 1: Figure S9).

EXO-depleted MSC-CM 25 wks injections did not rescue the PH and RVH associated with hyperoxia-induced BPD. The ratio between PAAT and PAET show significant decrease in BPD, DMEM:F12 and EXO-depleted MSC-CM 25 wks-injected groups as compared to RA, indicative of PAH (Fig. 4g). RV to LV ratio, indicative of RVH, was statistically significantly increased in BPD, DMEM:F12 and EXO-depleted MSC-CM 25 wks-injected groups as compared to RA (Fig. 4h). The data set of MSC-CM 25 wks injected in BPD mice, used earlier in this study, has been shown for comparison. Thus, we demonstrate that EXO-depleted MSC-CM 25 wks does not reverse hyperoxia-induced lung alterations and PH and RVH associated with hyperoxia-induced BPD, suggesting that the therapeutic action of MSC-CM resides in the EXO fraction.

### Human BPD tracheal aspirate and lung tissue of the BPD mouse model demonstrate elevated TSG-6

Our results demonstrate that administration of UC-MSC-CM EXO attenuates BPD pathology in lungs, heart and brain. To determine which factors in EXO are responsible for the recovery, we tested MSC-CM EXO 25 wks for the presence of an important immunomodulatory molecule, tumor necrosis factor α stimulated gene-6 (TSG-6). The anti-inflammatory activity has been directly demonstrated in a number of rodent models of inflammation including models of arthritis [36, 37], myocardial infarction [38], chemical injury to the cornea [39] and peritonitis [40]. We analyzed the TSG-6 RNA expression levels in human tracheal aspirates in patients who developed BPD and demonstrated significantly elevated levels as compared to non-BPD group (Additional file 1: Figure S10A). This clinical result corresponds to our mouse lung data, which demonstrates an increase in TSG-6 protein expression under pathological conditions in lung tissue of BPD mice as a protective response to increased inflammation (Additional file 1: Figure S10B). We found by Western blotting and its densitometric analysis that TSG-6 levels significantly increased in BPD and PBS-injected BPD mice lung tissues compared to RA (Additional file 1: Figure S10B). However, on administration of MSC-CM EXO in BPD mice, TSG-6 levels in their lungs decreased to that of RA levels. No statistically significant difference was seen in TSG-6 levels between BPD mice and BPD mice injected with vehicle PBS, thus suggesting that the increase in TSG-6 expression in BPD or PBS-injected BPD mice may be associated with inflammatory response for protection against hyperoxia-induced lung injury (Additional file 1: Figure S10B).

### TSG-6 detected in EXO fraction of MSC-CM 25 wks protects against pulmonary inflammation and reverses morphometric alterations associated with hyperoxia-induced lung injury

Western blotting was performed to check for the presence of TSG-6 in 25 wks GA MSCs, CM and EXO (Additional file 1: Figure S10C). Vehicle controls - DMEM:F12 and PBS - were also loaded. Specific TSG-6 signal at ~ 37 kDa (the size of TSG-6) was detected in 25 wks CM and EXO fractions (Additional file 1: Figure S10C). No signal was detected for DMEM:F12 and PBS fractions, which are devoid of any cells and cellular compartments, thus depicting presence of TSG-6 in the EXO fraction of MSC-CM 25 wks.

**Fig. 5:**
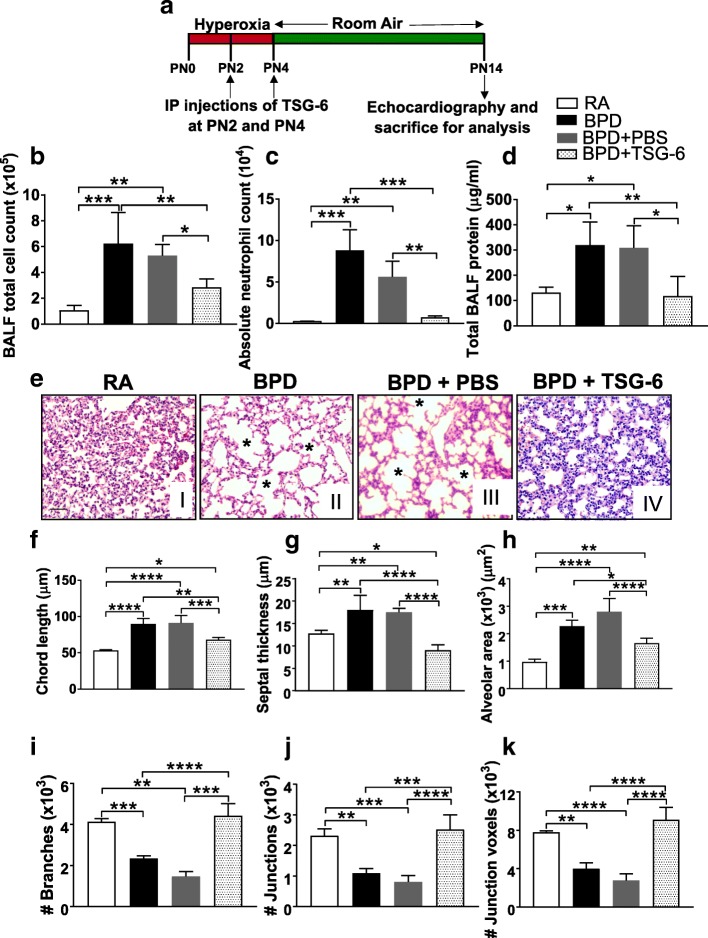
TSG-6 reverses hyperoxia-induced pulmonary inflammation and altered morphology in the BPD mice. **a** Schematic representation of BPD mouse model and TSG-6 injection regime. 5 μg of rhTSG-6 resuspended in total 100 μl of PBS was injected into each neonatal mouse at PN2 and PN4. **b-d** Histograms showing BALF total cell count (**b**), BALF absolute neutrophil count (**c**), total BALF protein (**d**) in RA, BPD, PBS-injected and TSG-6-injected BPD mice at PN14. **e** Representative images of lung histology with H&E stain from the four experimental groups, RA (I), BPD (II), BPD + PBS (III), BPD + TSG-6 (IV). *Asterisks* depict the increased alveolar simplification in the BPD and PBS-injected BPD mice as compared to RA. ×200 magnification, Scale bar: 50 μm. **f-k** Histograms showing the mean chord length (**f**), septal thickness (**g**), alveolar area (**h**), number of branches (**i**), junctions (**j**), junction voxels (**k**) in lungs of RA, BPD, PBS-injected, TSG-6-injected BPD mice. All values are expressed as mean ± SEM; four experiments, N = 3–8 mice per group; one-way ANOVA with Tukey’s post hoc correction; *p < 0.05; **p < 0.01; ***p < 0.001; ****p < 0.0001. *BALF* bronchoalveolar lavage fluid, *BPD* bronchopulmonary dysplasia, *CM* conditioned medium, *EXO* exosomes, *i.p.* intraperitoneal, *MSC* mesenchymal stem cell, *PAAT* pulmonary artery acceleration time, *PAET* pulmonary artery ejection time, *PBS* phosphate-buffered saline, *PN* postnatal, *RA* room air, *TSG-6* tumor necrosis factor alpha-stimulated gene-6

To investigate whether TSG-6, detected in MSC-CM EXO 25 wks, acts as one of the mediators in attenuation of BPD pathology, we injected recombinant human (rh) TSG-6 intraperitoneally in the BPD mouse model and studied its therapeutic effects. Administration of TSG-6 protein in BPD mice at PN2 and PN4 (Fig. 5a) significantly decreased the total cell count (Fig. 5b), infiltration of neutrophils (Fig. 5c) and protein leak (Fig. 5d) in the BALF of the TSG-6-injected group versus BPD, thus demonstrating decreased hyperoxia-induced alveolar-capillary leakage and preserved alveolar-capillary barrier in the lung.

The increase in mean chord length and septal thickness found in BPD was significantly ameliorated by TSG-6 treatment (Fig. 5e-g), depicting the therapeutic effect of TSG-6. Significant increase in alveolar area, observed in BPD group, was significantly reduced after TSG-6 injections in BPD mice (Fig. 5h). Interestingly, on TSG-6 administration, other lung morphological parameters like number of branches, junctions, junction voxels (Fig. 5i-k), triple points and quadruple points (Additional file 1: Figure S11A, B) showed statistically significant improvement in BPD mice. To summarize, TSG-6 treatment significantly improved pulmonary architecture in the BPD model, suggesting an important role of TSG-6 as a therapeutic molecule in hyperoxia-induced lung injury.

We also demonstrate that TSG-6 decreased the loss of peripheral pulmonary blood vessels in the BPD mouse (Additional file 1: Figure S11C, D). The immunofluorescence intensity of CD31 was significantly reduced in BPD, which reverted back to RA levels in TSG-6-injected groups, thus demonstrating decreased loss of peripheral blood vessels after TSG-6 administration.

### TSG-6 reverses BPD-associated cardiac and brain pathologies in the BPD mouse model

**Fig. 6:**
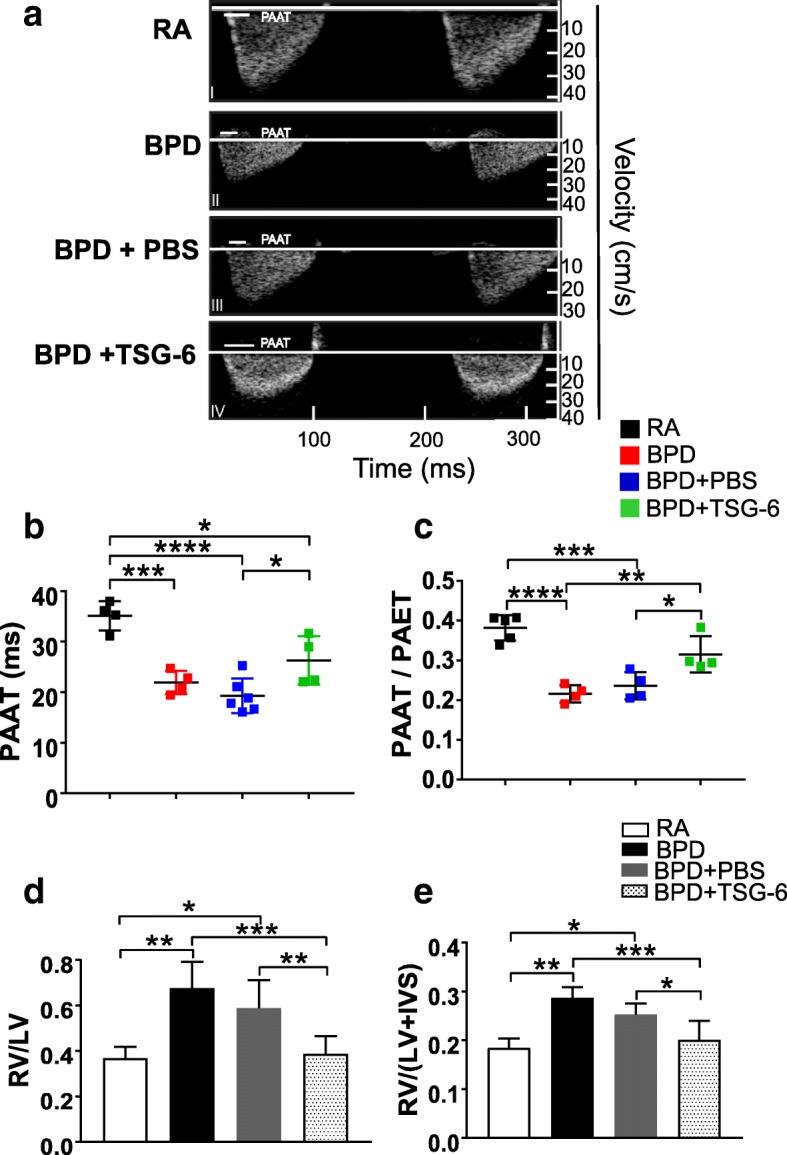
TSG-6 treatment corrects PH and RVH and attenuates hypomyelination and GFAP decrease in BPD mouse brain. **a** Echocardiography shows reversal of cardiac dysfunction on TSG-6 treatment in the BPD mouse model. The *white line* in the echocardiogram depicts PAAT values for specific group. **b** Graph demonstrating PAAT values from the four experimental groups at PN14. **c** Graph depicting PAAT/PAET ratio from the four experimental groups at PN14. **d** Histogram showing the RV to LV ratio in the four experimental groups. **e** Fulton’s index, [RV/(LV + IVS)], reflecting RV hypertrophy, in the four experimental groups at PN14. All values are expressed as mean ± SEM; four experiments, N = 3–8 mice per group; one-way ANOVA with Tukey’s post hoc correction; *p < 0.05; **p < 0.01; ***p < 0.001; ****p < 0.0001. *BALF* bronchoalveolar lavage fluid, *BPD* bronchopulmonary dysplasia, *CM* conditioned medium, *IVS* interventricular septa, *LV* left ventricular, *PBS* phosphate-buffered saline, *RA* room air, *RV* right ventricular, *TSG-6* tumor necrosis factor alpha-stimulated gene-6

PAAT/PAET values were significantly decreased in the BPD and PBS-injected groups, as compared to RA. However, treatment with TSG-6 significantly increased PAAT/PAET ratio, indicative of reversal of PH. No difference between the BPD and PBS-injected groups was observed, depicting specific attenuation after TSG-6 injections (Fig. 6a-c). RV/LV ratio was significantly increased and Fulton’s index in BPD mice was significantly decreased on TSG-6 treatment (Fig. 6d-e), depicting a reversal of RVH.

TUNEL assay demonstrated no significant difference in cell death in the olfactory bulb region between RA, BPD, PBS (vehicle)-injected and TSG-6 groups (Additional file 1: Figure S12A). However, TSG-6 treatment reduced cell apoptosis in forebrain and hindbrain (Additional file 1: Figure S12B, D). We found a trend towards decreased cell death in the TSG-6-injected versus PBS-injected group in forebrain and hind brain, which was not significant. We show statistically significant decrease in cell death in midbrain of TSG-6-treated pups (Additional file 1: Figure S12C), demonstrating that TSG-6 differentially attenuates cell death in the midbrain of BPD mice.

TSG-6 administration significantly increased MBP and GFAP expression in BPD brain (corpus callosum and hippocampus regions, respectively) (Additional file 1: Figure S12E-H), compared to BPD or PBS-injected groups, suggesting a dcrease in myelin loss and an increase in astrocyte formation.

### TSG-6 treatment decreases proinflammatory cytokines IL-6, TNF-α and IL-1β and cell death in lungs of the BPD mouse model

**Fig. 7:**
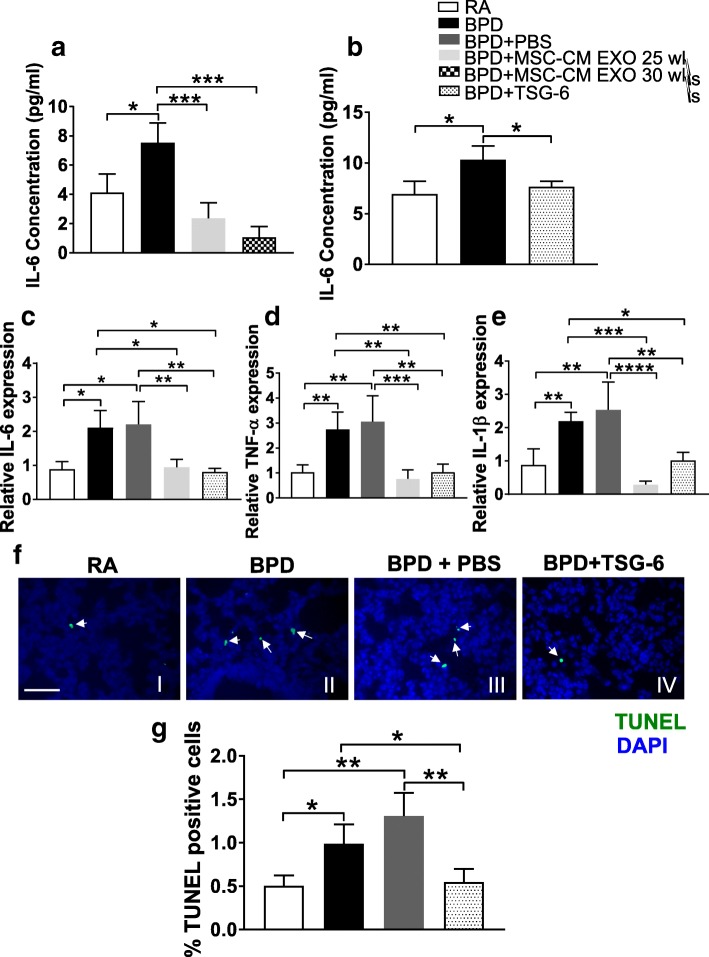
TSG-6 treatment decreases proinflammatory cytokines and cell death in lungs of the BPD mouse model. **a** IL-6 levels measured using ELISA in lung tissue of RA, BPD, MSC-CM EXO 25 wks- and MSC-CM EXO 30 wks-injected BPD mice at PN14. **b** IL-6 levels measured using ELISA in lung tissue of RA, BPD and TSG-6-injected BPD mice at PN14. **c-e** Pro-inflammatory cytokines levels in the lung tissues of RA, BPD and BPD mice injected with PBS, MSC-CM EXO 25 wks and TSG-6. Histogram showing relative expression of IL-6 (**c**), TNF-α (**d**) and IL-1β (**e**) as determined using real-time PCR. **f** Representative TUNEL immunofluorescence images of lung from the four experimental groups, RA (I), BPD (II), BPD + PBS (III), BPD + TSG-6 (IV). TUNEL-positive cells are labeled with FITC (*green*). The nuclei are counterstained with DAPI. *Arrows* depict TUNEL-positive dead cells in the field. Scale bar: 50 μm. **(g)** Histogram depicting the quantitative analysis of TUNEL-positive cells in the lungs of RA, BPD, PBS-injected and TSG-6-injected BPD mice. TUNEL-positive cells are expressed as a percentage, as described in Additional file 1: Methods. All values are expressed as mean ± SEM; 3–4 experiments, N = 3–5 mice per group; one-way ANOVA with Tukey’s post hoc correction; *p < 0.05; **p < 0.01; ***p < 0.001; ****p < 0.0001. *BPD* bronchopulmonary dysplasia, *CM* conditioned medium, *DAPI* 4,6-diamidino-2-phenylindole, *EXO* exosomes, *IL-6* interleukin-6, *IL-1β* interleukin 1 beta, *MSC* mesenchymal stem cell, *PBS* phosphate-buffered saline, *RA* room air, *TNF-α* tumor necrosis factor alpha, *TSG-6* tumor necrosis factor alpha-stimulated gene-6, *TUNEL* terminal deoxynucleotidyl transferase dUTP nick-end labeling

IL-6, a proinflammatory cytokine, has been shown to be elevated in different lung diseases [41]**.** Hyperoxia increases levels of IL-6 and causes increased lung cell death in newborn mice [20]. ELISA was performed to determine whether MSC-CM EXO treatment or TSG-6 administration decreases the levels of proinflammatory cytokine IL-6 in the lungs of BPD mice. We demonstrate that IL-6 levels were decreased in lung after MSC-CM EXO 25 wks/30 wks or TSG-6 treatment, indicating a decreased proinflammatory response (Fig. 7a-b). We demonstrate that the increased expression of IL-6 and other proinflammatory cytokines, such as TNF-α and IL-6, in BPD groups were significantly attenuated on treatment with MSC-CM EXO 25 and TSG-6 (Fig. 7c-e) as shown by real-time PCR.

We demonstrate that TSG-6 administration also decreased hyperoxia-induced cell death in the lungs in the BPD mouse. Percentage of TUNEL-positive dead cells were significantly increased in BPD, which reverted back to RA levels in TSG-6-injected groups (Fig. 7f-g). Decrease in cell death in the BPD lung on TSG-6 administration suggests that TSG-6 may partly prevent the lung injury induced by BPD in neonatal mice, probably via modulating the expressions of proinflammatory cytokines like IL-6, TNF-α and IL-1β in the lung tissue.

### Knockdown of TSG-6 in MSC-CM EXO significantly decreases therapeutic effects of MSC-CM EXO

**Fig. 8:**
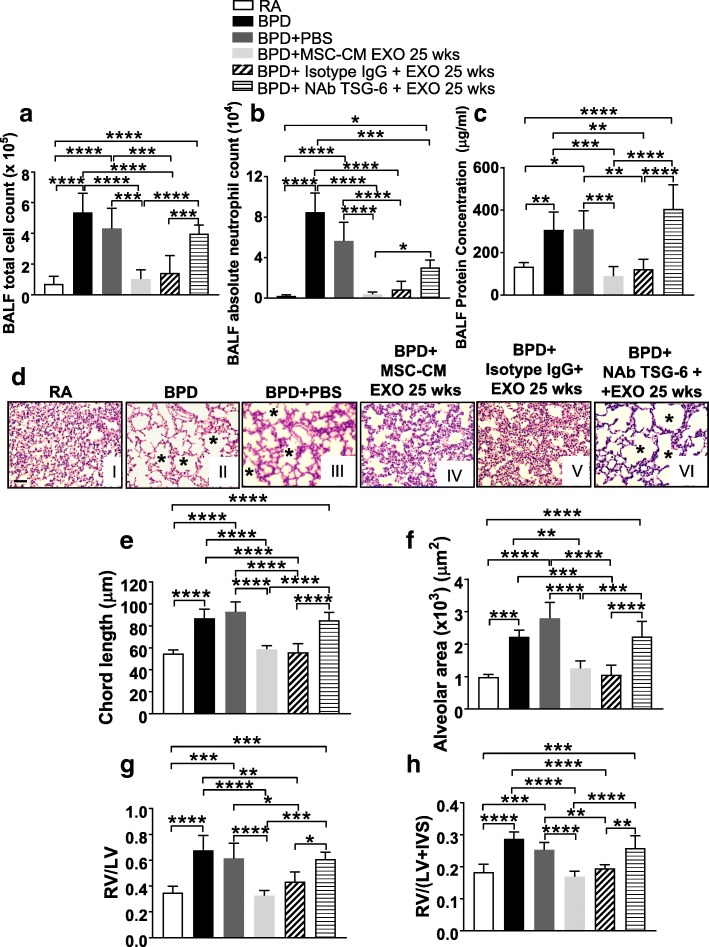
Administration of TSG-6 neutralizing antibody abrogates the therapeutic effects of MSC-CM EXO 25 wks. **a-c** Histogram showing BALF total cell count (**a**), BALF absolute neutrophil count (**b**), total BALF protein (**c**), in RA, BPD and PBS-injected, MSC-CM EXO 25 wks-injected, Isotype IgG + EXO 25 wks and NAb TSG-6 + EXO 25 wks-injected BPD mice at PN14. **d** Representative images of lung histology (H&E staining) from the six experimental groups, RA (I), BPD (II), BPD + PBS (III), BPD + MSC-CM EXO 25 wks (IV), BPD + Isotype IgG + EXO 25 wks (V), BPD + NAb TSG-6 + EXO 25 wks (VI). *Asterisks* depict the increased alveolar simplification in the BPD, PBS and NAb TSG-6 + EXO-injected BPD mice as compared to RA. ×200 magnification, Scale bar: 50 μm. **e-f** Histograms showing the mean chord length (**e**), alveolar area (**f**) in lungs of RA, BPD, PBS-injected, Isotype IgG + EXO 25 wks- injected and NAb TSG-6 + EXO 25 wks-injected BPD mice. **g-h** Histogram showing the RV to LV ratio (**g**) and Fulton’s index [RV/(LV + IVS)] (**h**), reflecting right ventricular hypertrophy at PN14. 5 μg of NAb resuspended in total 100 μl of PBS was injected into the neonatal mice at PN2 and PN4. RA, BPD, BPD + PBS and BPD + MSC-CM EXO 25 wks samples used for comparison in this analysis are from Figs. 1, 2 and 3. All values are expressed as mean ± SEM; six experiments, N = 3–8 mice per group; one-way ANOVA with Tukey’s post hoc correction; *p < 0.05; **p < 0.01; ***p < 0.001; ****p < 0.0001. *BALF* bronchoalveolar lavage fluid, *BPD* bronchopulmonary dysplasia, *CM* conditioned medium, *EXO* exosomes, *IVS* interventricular septa, *LV* left ventricular, *MSC* mesenchymal stem cell, *NAb* neutralizing antibody, *PBS* phosphate-buffered saline, *RA* room air, *RV* right ventricular, *TSG-6* tumor necrosis factor alpha-stimulated gene-6

To analyze the role of TSG-6 as a therapeutic mediator, loss of the function of TSG-6 was performed by TSG-6 knockdown using TSG-6 neutralizing antibodies (NAb) or TSG-6 siRNA. Intraperitoneal injection of TSG-6 NAb was performed in BPD mice, spaced between MSC-CM EXO 25 wks injections, at PN1, PN3 and PN5. MSC-CM EXO 25 wks was administered at PN2 and PN4. Pups were transferred to RA from PN4 to PN14 followed by echocardiography and collection of tissue for the study. Administration of TSG-6 NAb in BPD significantly increased the total cell count (Fig. 8a), infiltration of neutrophils (Fig. 8b) and protein leak (Fig. 8c) in the BALF of TSG-6 NAb-injected group versus RA or Isotype IgG control, thus demonstrating increased hyperoxia-induced alveolar-capillary leakage and disrupted alveolar-capillary barrier in the lung. There was no change in the percentages of BALF macrophages among different groups in NAb TSG-6 EXO 25wks-injected BPD mice **(**Additional file 1: Figure S13A). The mean chord length and alveolar area (Fig. 8d-f) was comparable to that of BPD and significantly more than RA, depicting significant hyperoxia-induced damage even after EXO treatment. PAAT values were significantly decreased in the BPD and PBS-injected groups, as compared to RA. There was no significant difference between RA and MSC-CM EXO 25 wks-treated BPD mice. However, treatment with NAb TSG-6 + EXO 25 wks significantly decreased PAAT value, indicative of PH. No difference between BPD and NAb TSG-6 -injected groups was observed (Additional file 1: Figure S13B). RV/LV ratio and Fulton’s index was significantly increased on NAb TSG-6 + EXO 25 wks treatment (Fig. 8g-h), depicting BPD pathology of the heart. The control Isotype IgG group was not statistically different from the RA group, but there was a statistically significant difference between the Isotype IgG control group or MSC-CM EXO 25 wks and NAb TSG-6 + EXO 25 wks group, thus demonstrating a specific decrease in the therapeutic effects of the EXO on TSG-6 knockdown.

Twenty-five weeks GA MSCs were transfected with TSG-6 siRNA, and TSG-6 knockdown efficiency was calculated. We obtained 70–75% knockdown of TSG-6 in MSCs, when compared to the control group comprising MSCs transfected with scr siRNA (Additional file 1: Figure S13C-D). EXO was isolated from TSG-6siRNA-transfected MSC-CM and injected in the mouse model of BPD to analyze the effect of TSG-6 siRNA EXO 25 wks on different BPD parameters.

**Fig. 9:**
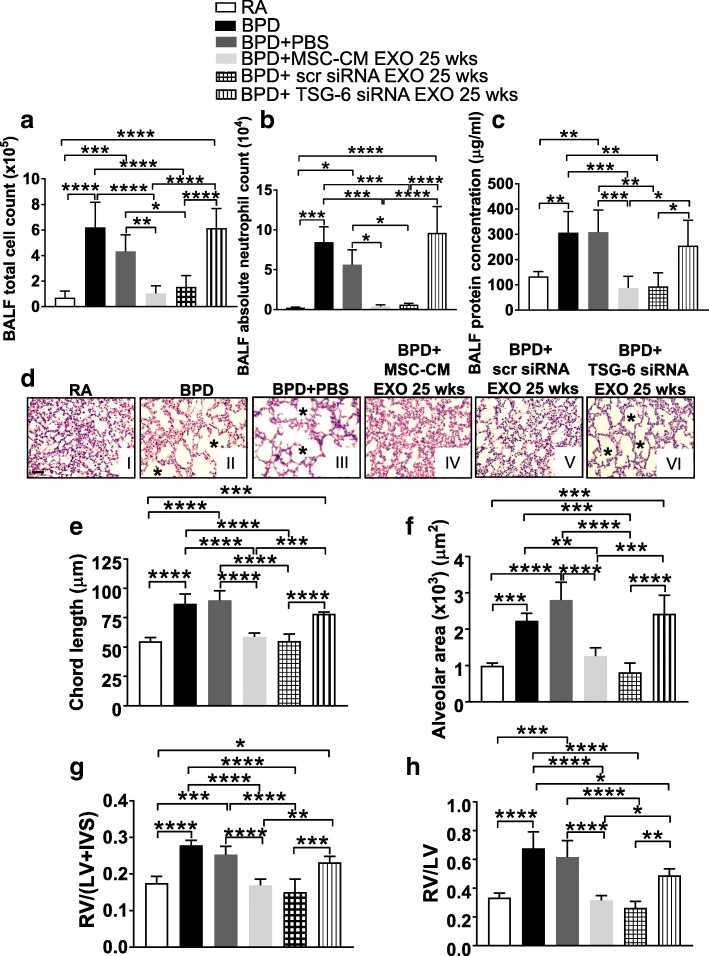
Administration of EXO isolated from TSG-6 siRNA knockdown MSCs did not suppress hyperoxia-induced inflammation and associated BPD pathology. **a-c** Histogram showing BALF total cell count (**a**), BALF absolute neutrophil count (**b**), total BALF protein (**c**), in RA, BPD and PBS-injected, MSC-CM EXO 25 wks-injected, scr siRNA EXO 25 wks and TSG-6 siRNA EXO 25 wks-injected BPD mice at PN14. **d** Representative images of lung histology (H&E staining) from the six experimental groups, RA (I), BPD (II), BPD + PBS (III), BPD + MSC-CM EXO 25 wks (IV), BPD + scr siRNA EXO 25 wks (V), BPD + TSG-6 siRNA EXO 25 wks (VI). *Asterisks* depict the increased alveolar simplification in the BPD, PBS and TSG-6 siRNA EXO 25 wks-injected BPD mice as compared to RA. ×200 magnification, Scale bar: 50 μm. **e-f** Histograms showing the mean chord length (**e**), alveolar area (**f**) in lungs of RA, BPD, PBS-injected, scr siRNA EXO 25 wks-injected and TSG-6 siRNA EXO 25 wks-injected BPD mice. **g-h** Histogram showing the RV to LV ratio (**g**) and Fulton’s index [RV/(LV + IVS)] (**h**), reflecting right ventricular hypertrophy at PN14. RA, BPD, BPD + PBS and BPD + MSC-CM EXO 25 wks samples used for comparison in this analysis are from Figs. 1, 2 and 3. All values are expressed as mean ± SEM; six experiments, N = *3–*8 mice per group; one-way ANOVA with Tukey’s post hoc correction; *p < 0.05; **p < 0.01; ***p < 0.001; ****p < 0.0001. *BALF* bronchoalveolar lavage fluid, *BPD* bronchopulmonary dysplasia, *CM* conditioned medium, *EXO* exosomes, *IVS* interventricular septa, *LV* left ventricular, *MSC* mesenchymal stem cell, *PBS* phosphate-buffered saline, *RA* room air, *RV* right ventricular, *scr siRNA* scrambled small interfering ribonucleic acid, *TSG-6* tumor necrosis factor alpha-stimulated gene-6

**Fig. 10:**
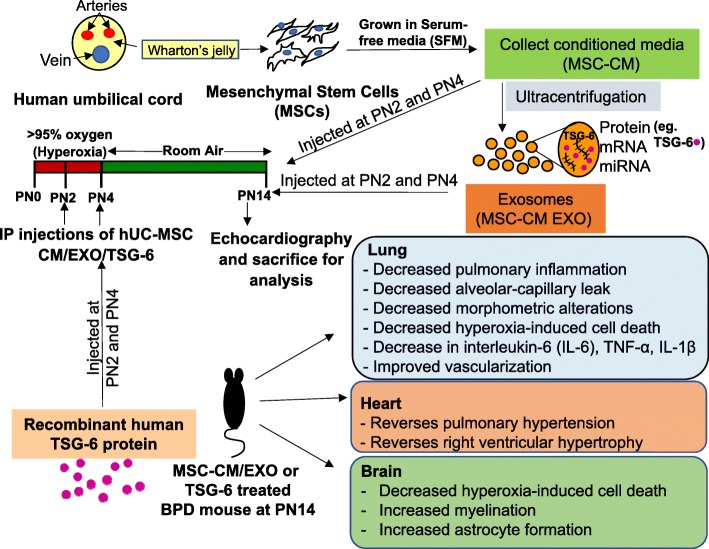
Schematic representation of MSC-CM/EXO/TSG-6 treatment regimen and outcomes in the mouse model of BPD. BPD pathology is reversed in the pups injected with secretome of hUC-derived MSCs. This therapeutic effect is mediated, at least in part, by exosomal factor TSG-6. *BPD* bronchopulmonary dysplasia, *CM* conditioned medium, *EXO* exosomes, *hUC* human umbilical cord, *i.p.* intraperitoneal, *MSC* mesenchymal stem cell, *PN* postnatal, *TSG-6* tumor necrosis factor alpha-stimulated gene-6

Administration of TSG-6 siRNA EXO 25 wks into BPD mouse significantly increased the total cell count (Fig. 9a), absolute neutrophil counts (Fig. 9b) and protein leak (Fig. 9c) in the BALF of TSG-6 siRNA EXO 25 wks-injected group versus RA or scr siRNA control. There was no change in the percentages of BALF macrophages among different groups in TSG-6 siRNA EXO 25 wks-injected BPD mice (Additional file 1: Figure S13E). The mean chord length and alveolar area (Fig. 9d-f) was comparable to that of BPD and significantly more than RA, depicting significant hyperoxia-induced damage. PAAT values were significantly decreased in the BPD and PBS-injected groups, as compared to RA. There was no significant difference between RA and MSC-CM EXO 25 wks-treated BPD mice. However, treatment with TSG-6 siRNA EXO 25 wks significantly decreased PAAT value, indicative of PH. No difference between BPD and TSG-6 injected groups was observed (Additional file 1: Figure S13F). RV/LV ratio and Fulton’s index was significantly increased on TSG-6 siRNA EXO 25 wks treatment (Fig. 9g-h), depicting BPD pathology of the heart. The control scr siRNA EXO 25 wks group was not statistically different from the RA group, but there was a statistically significant difference between the scr siRNA control group or MSC-CM EXO 25 wks and TSG-6 siRNA EXO 25 wks group, thus demonstrating specific loss in the therapeutic effects of the EXO on TSG-6 knockdown. Thus, we see that knockdown or silencing of TSG-6 in the administered MSC-CM EXO results in loss of therapeutic activity, whereas administering exogenous TSG-6 rescues the therapeutic activity. To summarize, our results demonstrates that the therapeutic effects of the secretome of UC MSCs resides in the EXO fraction, and specifically, TSG-6 is one of the mediators in the 25 wks GA MSC-CM EXO, responsible for the attenuation of BPD pathology (Fig. 10).

## Discussion

Determining the most appropriate cell type and tissue source is crucial for successful clinical translation of cell-based therapies for protection against BPD. UC-WJ represents an ethically non-controversial, clinically relevant and easily accessible source of potent stem cells [42]. MSCs derived from UC exhibit biological advantages over adult adipose tissue-derived or bone marrow-derived MSCs, showing low immunogenicity, higher proliferative capacity, increased secretion of chemokines, proinflammatory proteins and growth factors and better neuroprotection efficacy [43–46]. Several lines of evidence suggest that the therapeutic action of MSCs is due to its paracrine effect rather than through cell replacement [47–49]. Different sources of secretome have been used including bone marrow-derived MSCs [10] in contrast to our source of secretome isolated from preterm UC-MSCs WJ. However, not much is known about the efficacy of preterm MSC secretome and its paracrine factors in the treatment of BPD at a developmentally appropriate window. Several animal models have been developed and refined, representing the pathological pulmonary characteristics of the lungs of human neonates with BPD. The mouse model of BPD is most common due to relatively short gestational age, allowing studies on different developmental stages of lung. The saccular stage of murine lung development occurs between E17 and PN4, therefore, full-term mouse represents a developmental stage resembling that of a human preterm neonate between 24 and 28 weeks. Our results are described in the context of the experimental model we used [19, 20, 22–26], where we show the model of severe BPD (oxygen exposure in the saccular stage, from birth till PN4) as compared to the models of hyperoxia-induced acute lung injury (HALI), where exposure to 60–100% oxygen is up to 2–4 weeks in mouse, throughout saccular and alveolar stages [29], which corresponds to the developmental stages extending from preterm to adolescence in humans. These are important factors when assessing translational significance, as modeling needs to be developmentally appropriate to mimic human BPD as closely as possible [19 16]. Additionally, although full-term mouse lungs, in the saccular stage, are competent for proper gas exchange, human preterm neonates often require supplemental oxygen and surfactant administration. The model used in this study could represent a human preterm neonate who has been adequately administered prenatal steroids (which results in lung maturation) and is exposed to a high concentration of supplemental oxygen [21].

As higher number of circulating CD34+ hematopoietic stem and progenitor cells have been reported in extremely preterm neonates compared to the full-term neonates [18], we chose to isolate MSCs from 25 and 30 wks GA UC to see whether CM or EXO from earlier GA MSCs demonstrate therapeutic effects on BPD pathology. We demonstrate that MSC-CM or EXO from both 25 and 30 wks GA UC attenuated BPD pathology even after the injury has commenced. Detailed lung morphometric analyses reveal that MSC-CM or EXO from 25 wks GA UC had statistically significant improvement in some parameters of BPD lung morphology compared to 30 wks GA UC. This effect was more pronounced in EXO fractions. This study demonstrates for the first time that MSC-CM or EXO from earlier GA UC is a suitable source for cell-free therapy for BPD. To our knowledge, this is the first quantitative assessment of effects of preterm UC-MSC-CM and EXO in vivo in terms of BPD pathology in multiple organs including lung, heart and brain. We chose early time points for intervention, which corresponds to the early phase of inflammation, suggested to be the best anti-oxidative and therapeutic time window of stem cell transplantation in BPD [50, 51]. As only limited volume can be injected in the neonatal pups, we concentrated the therapeutic paracrine mediators in the secretome before administration. For injections, the CM was concentrated tenfold. Our results demonstrate that the extent of correction and survival in a BPD model using EXO fraction is equivalent to that of a CM fraction isolated from the UC-MSCs. CM and EXO fractions were obtained from MSCs which were grown in SFM for 24 h, after washing the monolayer with PBS, to ensure complete elimination of FBS contamination in the collected CM or EXO.

Our previous study demonstrates that preterm hUC MSC act as a potential therapeutic agent via paracrine mechanism [52]; however, no significant therapeutic effect was observed when human dermal fibroblasts (HDF-control cells) were injected in our BPD model. Interestingly, Kourembanas’ group have also reported no protection after injection with control cells, HDF or HDF-EXO in the BPD mouse, thus showing the specificity of the MSCs and its EXO for therapeutic use [17]. We demonstrate that DMEM:F12/PBS control injections do not have any significant protective effect in BPD mice. Earlier studies on long-term (6 months) assessment of MSC-CM therapy show no adverse lung effects on lung function and structure, when MSC-CM was injected in RA control animals, thus showing that the CM is safe to use for BPD therapy [53]. WJMSC-EXO administration had no effect on non-injured lungs as measured by mean linear intercept of RA mice and RA mice treated with WJMSC-EXO [17]. Kourembanas’ group had previously found that EXO-depleted CM had no effect in their animal model of BPD [29]. Furthermore, we demonstrate that EXO-depleted MSC-CM 25 wks, when injected in our BPD mouse model, does not have any protective effects, establishing that the protective factors are present in the EXO fraction.

Secreted EXO are an attractive and quite plausible means for MSCs to regulate multiple pathways and produce a robust therapeutic effect in vivo [54–56]. These packaging vesicles in the MSC-CM, compared with cells, are less immunogenic, more stable and can be stored frozen with no loss of activity, and have no risk of aneuploidy following in vivo allogeneic administration. EXO can cross the blood–brain barrier, making them particularly attractive as a new tool therapies for neurodegenerative diseases. Delivering EXO into the CNS using intravenous, or intranasal, or intraperitoneal routes avoids the need for neurosurgical interventions [57].

TSG-6, a 35–39 kDa-secreted anti-inflammatory protein produced by many cell types in response to inflammatory mediators like TNF-α and IL-1β, is one of the key factors in the immunosuppressive properties of MSCs [38–40, 58]. Microarray analysis show TSG-6 as one of the most highly upregulated transcripts in lung-lodged transplanted MSCs [38]. Previous studies report that TSG-6, via binding to CD44, induces a phenotypic shift in macrophages from proinflammatory M1 to anti-inflammatory M2 type that protects the mice against lung inflammation and injury induced by endotoxin lipopolysaccharide (LPS) [32, 59]. TSG-6 can play a role by inhibiting components in the inflammation network of proteases [58], suppressing neutrophil migration into the site of inflammation [60]. RNA sequencing and its gene ontology show that there are 41 genes specifically induced by WJMSC-EXO treatment, few of them are likely associated with extracellular matrix and structural organization [17]. TSG-6 binds to hyaluronan (HA) that is essential for stabilizing and remodeling the extracellular matrix (ECM) during ovulation and inflammatory disease processes such as arthritis [61]. MSCs seem to have relevant neuroprotective properties in experimental injury models of the brain [62, 63], which is suggested to be mediated by TSG-6. An earlier study, based on antibody protein array and ELISA, to examine protein levels of cytokines released by MSC-CM under serum-starved conditions show that the TSG-6 levels increased dramatically [64]. Based on above observations, we chose to analyze TSG-6 in the EXO fraction as one of the mediators of therapeutic actions of MSC-CM.

TSG-6 is detected in a physiological and pathological context associated with inflammation and tissue remodeling [36, 65]. We report high levels of TSG-6 in the lungs of BPD mouse, suggesting that BPD is associated with elevated levels of TSG-6. This increase in TSG-6 levels could be a temporal and developmental-specific protective response. We detected TSG-6 protein in the CM and in EXO fractions of 25 wks GA UC indicating that TSG-6 is secreted by MSCs and TSG-6 by itself or in combination with other mediators could yield new therapeutic options. We demonstrate that i.p. administration of hUC-MSC secretome significantly attenuates neonatal hyperoxia-induced BPD pathology, in part, via the secretome-associated factor TSG-6.

TSG-6 is an immunomodulatory molecule, which has been shown to be increased in pathological conditions in response to increased inflammation. We report that TSG-6 levels are highly elevated in the lungs of BPD mouse model, suggesting that BPD is associated with elevated levels of TSG-6. An earlier study, based on antibody protein array and ELISA, to examine protein levels of cytokine released by MSC-CM under serum-starved conditions show that the releasing of TSG-6 increased most dramatically [64]. In this study, we also found that TSG-6 is required for protection against hyperoxia-induced injury in lungs, heart and brain. Therefore, we hypothesize that the observed elevated TSG-6 levels in lungs during hyperoxia-induced lung injury could be a temporal and developmental-specific response from the cells as a defense mechanism or protection against the hyperoxia-induced inflammatory conditions. This suggests that TSG-6 is part of a negative feedback loop in the inflammatory response. Physiologically, it appears that one of the functions of TSG-6 is to counteract the inflammatory effects of TNF-a and IL-1 [66]. Silencing of TSG-6 in the administered MSCs resulted in loss of therapeutic activity, whereas, administration of exogenous TSG-6 resulted in replication of therapeutic activity, thus showing that the reduction in TSG-6 in EXO-injected BPD mice show improvement in the inflammatory status of the BPD mice.

Another possibility is that the elevated TSG-6 in BPD (a pathological condition) is because of enhanced inflammation reaching its threshold to the therapeutic level once the MSC-CM/EXO treatment is done. Once it reaches this level, TSG-6 level decreases due to a negative feedback response. This concept of “sufficiency” (reaching a maximal level to generate a “protective” response) and enhancement of this level to achieve an additional protective effect has been recognized in our mouse model of hyperoxia-induced BPD, in terms of autophagic flux [19].

PH is triggered in BPD by inflammation and endothelial dysfunction, and greatly increases the mortality in BPD patients [2]. Earlier studies have indicated a therapeutic benefit from bone marrow-derived MSCs in improving respiratory function in animal models of PH [67, 68]. We used non-invasive high-resolution echocardiography, a useful technique in translating the findings from preclinical evaluations to clinical meaningful conclusions, to evaluate cardiovascular function and changes after MSC-CM/EXO or TSG-6 injections in a BPD mouse model. We show that BPD and associated PH can be reversed with UC-MSC-CM/EXO treatment. EXO and TSG-6 treatment decreases loss of peripheral blood vessels and restrains the inflammation response, thus improving heart function after hyperoxia-induced lung injury. Interestingly, our result corresponds with the improved pulmonary function testing results of hyperoxia-induced lung injury on full-term MSC-EXO treatment reported earlier [17].

The preterm period in humans and early postnatal developments in rodents is the time of active oligodendrogenesis, myelination and axonal organization in the developing subcortical white matter. Neonatal hyperoxia exposure in the mouse disrupts development of the white matter by delaying the maturation of oligodendroglial lineage [69, 70]. Hyperoxia exposure has also been found to disrupt axon-oligodendrocyte integrity in the subcortical white matter [71]. Astrocytes promote axonal growth and deliver essential neurotrophic factors under both physiological and pathophysiological conditions. Loss and/or abnormal function of glial cells can contribute to neurodegeneration [72, 73]. This study suggests an important role of oligodendrocytes and astrocytes in BPD pathology. MSCs have neuroprotective properties in experimental injury models of the brain [62, 63, 74]. Another study demonstrates that administration of neural stem cells into PN2 neonatal brain shows a shift toward differentiation into oligodendrocytes [75]. However, the molecular mechanism underlying the neuroprotective actions of EXO is not well understood. We demonstrate that administration of MSC-CM EXO at early postnatal days reverses hypomyelination and increases astrocytes indicating the potential of MSC secretome for the treatment of neurological and neurodegenerative disease. Our study suggests that EXO protein, TSG-6, in part, plays an important role in neuroprotection in the BPD mouse model.

We have shown before that hyperoxia (95% oxygen) exposure in newborn mice increases levels of IL-6 and causes increased lung cell death [76]. This is in accord with our earlier observation that exposure to hyperoxia in a developmentally appropriate cell culture model leads to enhanced IL-6 release and cell death [77]. Furthermore, levels of IL-6, TNF-α and IL-1β are shown to be significantly increased in tracheal aspirates of premature neonates with an adverse outcome (BPD/death) [78–80]. We demonstrate that administration of MSC-CM EXO or TSG-6 decreases the proinflammatory cytokine IL-6 levels in the BPD mice. Other proinflammatory cytokines like TNF-α and IL-1β also show decreased levels in lung tissue on administration of MSC-CM EXO or TSG-6. Decrease in cell death in the BPD lung on TSG-6 administration suggests that TSG-6 may partly prevent the lung injury induced by BPD in neonatal mice probably via modulating the expressions of proinflammatory cytokines such as IL-6 in the lung tissue. Further research is required to understand the mechanism of cell death in the individual cell types of the lung.

Our study provides an important step toward the preclinical development of a cell-free therapeutic from MSCs that might be effective in preterm human infants at risk for developing BPD. Nevertheless, there are limitations of this study that need to be addressed before translation of the therapy to the clinical setting. More details are required to identify other critical components of EXO, be they protein, lipid or nucleic acid species, which are involved in preservation of lung growth and function in experimental BPD. Only one dose of MSC-CM EXO/TSG-6 was selected for these studies based on earlier studies and there is no dose–response data. More preclinical studies on larger animals are needed to standardize and identify a range of doses and timing for the intervention along with the functional assays.

Although we obtained EXO-enriched fraction using the differential centrifugation protocol, as characterized by its morphology, particle number and protein content, more uniform standardized EXO production methods are required for regulatory requirement and complete characterization of the purified fraction. It is interesting that we found similar therapeutic effects of preterm MSC-CM EXO in our mouse model of BPD on administrating EXO purified by differential centrifugation when compared to those purified by density gradient centrifugation [17]. We did not show any therapeutic effect with the 25 wks GA EXO-depleted MSC-CM fraction in our BPD model, suggesting that any contaminating proteins in EXO preparations could be inert excipients from the pharmacological standpoint [81]. Thus, this preparation could be used for the bench side work. However, for large-scale clinical trials, optimal purity and consistency of MSCs will be important, which will help in dosage calculations and determination of the comprehensive molecular and functional characterization of the active MSC EXO factors [82]. More mechanistic information is needed to better understand how preterm MSC-derived EXO or TSG-6 work as a preventive or early therapeutic approach for BPD. This will enable us to determine the best clinical candidates with BPD for MSC-derived CM/EXO/TSG-6 therapy.

Overall, our results suggest that a comprehensive strategy involving MSC secretome or TSG-6 administration could benefit in amelioration of lung diseases, particularly BPD and its associated pathologies affecting the heart and the brain. The present study gives an important lead for development of cell-free therapy for BPD and other lung injury conditions.

## Conclusions

The current study demonstrates for the first time that administration of preterm hUC-derived MSC-CM EXO improves BPD pathology of lung, heart and brain and reduces inflammation, at least in part, via exosomal factor TSG-6. TSG-6, detected in the EXO fraction, on administration decreases lung inflammation, alveolar-capillary leak, altered morphometry and cell death in the BPD mouse model. This was accompanied by an improvement in PH, pulmonary vascular remodeling, and decreased brain pathology. Moreover, the therapeutic effects of MSC-CM EXO were abrogated by TSG-6 knockdown in the MSCs using TSG-siRNA or by injecting TSG-6 NAb in the BPD mice along with the treatment with EXO. This loss in therapeutic action was not observed by scrambled siRNA or Isotype control, respectively, indicating an important role of TSG-6 in the treatment of hyperoxia-induced lung injury, BPD. The proinflammatory cytokines IL-6, TNF-α and IL-1β were decreased in EXO or TSG-6-treated BPD mice suggesting its modulatory role in lung injury. These findings suggest TSG-6 as one of the important protective mediator in therapies for BPD. This result may provide a novel therapeutic approach for the treatment of BPD and probably other pulmonary and neurological disorders.

## Additional file

Additional file 1: Supplementary experimental procedures. **Table S1.** Comparison of different parameters in the exosome dose. **Table S2.** Clinical data for BPD and No BPD (*n* = 10). **Figure S1.** Preterm hUCs MSCs, but not fibroblasts, improve the BPD pulmonary phenotype. i.p. injections of MSCs from preterm (32 weeks) hUCs, in our mouse BPD model, at PN2 and PN4 showed improvement in lung architecture (at PN14), as evidenced by decreased chord length compared to the BPD mice. Human primary dermal fibroblast (HDF) cells, injected as control cells in the BPD mice, demonstrated no difference as compared to BPD mice. All values are expressed as mean ± standard deviation (SD); four experiments, *N* = 3 mice per group; one-way ANOVA with Tukey’s post hoc correction; *p < 0.05; **p < 0.01; ***p < 0.001. **Figure S2.** Isolation and characterization of preterm UC-MSCs **(A)** UC-MSCs from 25 and 30 wks gestational age UC, after Passage 1, 2 days (I, III) and 5 days (II, IV) in culture respectively. The cells were adherent to the culture flasks and displayed fibroblast-like morphology. **(B)** FACS characterization of UC-MSCs. UC-MSCs were positive for CD105, CD73 and CD90 and were negative for HLA-DR, CD19 and CD14. **(C)** Differentiation of UC-MSCs into adipocytes (Oil Red O staining), osteocytes (Alizarin Red S staining) and chondrocytes (Alcian Blue staining). **Figure S3. (A-F).** Isolation and identification of hUC MSC-CM EXO **(A)** Transmission electron micrograph (TEM) of hUC-MSC-EXO 25 wks (I) and 30 wks (II) depicts intact exosomes with a diameter of 40–120 nm. Scale bar: 100 nm. **(B)** Particle number and size of isolated exosomes by nanosight nanoparticle tracking analysis. Representative plot of the 25wks EXO and 30 wks EXO, samples 1:50 and 1:20 diluted, respectively, for the analysis. **(C)** Dot blot of UC-MSC-CM 25 wks (I), UC-MSC-CM 25 wks EXO (II), UC-MSC-CM 30 wks (III), UC-MSC-CM EXO 30 wks (IV), show positive signal for exosome-specific antibody CD63 while no signal is detected in negative controls - DMEM:F12 media alone (V) or PBS (VI). **(D)** Dot blot of exosomal fractions [hUC-MSC-CM EXO 25 wks (II), hUC-MSC-CM EXO 30 wks (IV)] did not show any signal after probing with cytoskeletal membrane antibodies (TGN 48), which was otherwise detected in CM fractions [from 25 wks GA UC (I) and 30 wks GA UC (III)] confirming the purity of the isolated exosomal fraction. No signal was detected in negative controls - DMEM:F12 (V) and PBS (VI). **(E)** Detection of CD81 in UC-MSCs, MSC-CM and MSC-CM EXO from 25 wks GA and 30 wks GA by Western blotting. Specific signal (25 kDa) was detected for CD81 in all fractions containing exosomes. No signal was seen in the loading controls, DMEM:F12 and PBS. Exosomal protein CD81 was also not detected in the EXO-depleted MSC-CM. **(F)** Detection of exosomal protein Alix-1 (97 kDa) in exosomes derived from 25 wks GA hUC MSCs by Western blotting. **Figure S4. (A)** Percentage of macrophages in BALF. Histogram depicting the percentage of macrophages in the BALF of RA, BPD, DMEM:F12 or PBS-injected, MSC-CM or EXO (25 wks-injected, 30 wks-injected) BPD mice at PN14. **(B-C)** Number of triple points and quadruple points. Histogram depicting number of triple points **(B)** and quadruple points **(C)** in lungs of RA, BPD, DMEM:F12 or PBS-injected, MSC-CM or EXO (25 wks-injected, 30 wks-injected) BPD mice at PN14. **(D-E)** Decreased hyperoxia-induced cell death in lungs of hUC MSC-CM-treated BPD pups. **(D)** Representative TUNEL immunofluorescence images of lung from the five experimental groups, RA (I), BPD (II), BPD + DMEM:F12 (III), BPD + MSC-CM 25 wks (IV), BPD + MSC-CM 30 wks (V). The nuclei are counterstained with DAPI. *Arrows* depict TUNEL-positive dead cells (*green*) in the field. Scale bar: 50 μm. **(E)** Histogram demonstrating the quantitative analysis of TUNEL-positive cells, expressed as a percentage, as described in Additional Information methods. All values are expressed as mean ± SEM; five experiments, *N* = 3*–*5 mice per group; one-way ANOVA with Tukey’s post hoc correction; *p < 0.05; **p < 0.01; ***p < 0.001; ****p < 0.0001. **Figure S5.** Decreased hyperoxia-induced cell death in lungs of hUC MSC-CM EXO-treated BPD pups. **(A)** Representative TUNEL immunofluorescence images of lung from the five experimental groups, RA (I), BPD (II), BPD + PBS (III), BPD + MSC-CM EXO 25 wks (IV), BPD + MSC-CM EXO 30 wks (V). The nuclei are counterstained with DAPI. *Arrows* depict TUNEL-positive dead cells (*green*) in the field. Scale bar: 50 μm. **(B)** Histogram demonstrating the quantitative analysis of TUNEL-positive cells, expressed as a percentage, as described in Additional Information methods. **(C)** Representative immunofluorescence images of lung from the five experimental groups, RA (I), BPD (II), BPD + PBS (III), BPD + MSC-CM EXO 25 wks (IV), BPD + MSC-CM EXO 30 wks (V) stained with cleaved caspase-3. The nuclei are counterstained with DAPI. *Arrows* depict TUNEL-positive dead cells (*red*) in the field. Scale bar: 50 μm. **(D)** Histogram demonstrating the quantitative analysis of cleaved caspase-3 positive cells, expressed as a percentage, as described in Additional Information methods. All values are expressed as mean ± SEM; five experiments, *N* = 3–5 mice per group; one-way ANOVA with Tukey’s post hoc correction; *p < 0.05; **p < 0.01; ***p < 0.001; ****p < 0.0001. **Figure S6.** MSC-CM EXO rescue hyperoxia-induced loss of peripheral pulmonary blood vessels **(A)** Representative images of lung sections stained with endothelial cell marker CD31. *Arrows* point to CD31 staining (*green*) in the blood vessels in the lungs of PN14 mice. The cells were counterstained with DAPI for nuclear staining. **(B)** Quantitative analyses of mean pixel intensity of CD31 immunofluorescence at PN14 in RA, BPD, PBS-injected and MSC-CM-EXO-injected BPD mice groups. Scale bar: 100 μm. All values are expressed as mean ± SEM; five experiments, N = 3–5 mice per group; one-way ANOVA with Tukey’s post hoc correction; *p < 0.05; **p < 0.01. Scale bar: 50 μm. **Figure S7.** hUC MSC secretome treatment decreases hyperoxia-induced cell death in the brain of the BPD mice. **(A-D)** Histogram demonstrating the quantitative analysis of TUNEL-positive cells, expressed as a percentage in the olfactory bulb **(A)**, forebrain region **(B),** midbrain region **(C)** and hindbrain **(D)** region of PN14 mouse brain after MSC-CM treatment. **(E-H)** Histogram demonstrating the quantitative analysis of TUNEL-positive cells, expressed as a percentage in the olfactory bulb **(E)**, forebrain region **(F),** midbrain region **(G)** and hindbrain **(H)** region of PN14 mouse brain after MSC-CM EXO treatment. All values are expressed as mean ± SEM; five experiments, *N* = 3–4 mice per group; one-way ANOVA with Tukey’s post hoc correction; *p < 0.05; **p < 0.01; ***p < 0.001; ****p < 0.0001. **Figure S8.** hUC MSC-CM EXO-treated pups decreased loss of myelination and increased GFAP expression in the brain of BPD mice. **(A)** Representative immunofluorescence images of MBP staining, marker for myelin, in RA, BPD, vehicle (PBS)-injected BPD mice, MSC-CM EXO 25 wks and MSC-CM EXO 30 wks-injected BPD mice (I, II, III, IV, V), to quantitate myelin loss in BPD and vehicle (PBS)-injected BPD mice (II, III) and restoration of myelination after MSC-CM EXO 25 wks (IV) and MSC-CM EXO 30 wks (V) injections. **(B)** Representative immunofluorescence images of GFAP staining in RA, BPD, PBS + BPD and MSC-CM EXO 25 wks and MSC-CM EXO 30 wks-injected BPD mice (I, II, III, IV, V). ×400 magnification, Scale bar: 50 μm **(C-D)** Histogram showing quantitation of MBP expression **(C)** and GFAP expression **(D)** in RA, BPD, BPD + PBS, BPD + MSC-CM EXO 25 wks and BPD + MSC-CM EXO 30 wks groups. All values are expressed as mean ± SEM; five experiments, *N =* 3–4 in each group; one-way ANOVA with Tukey’s post hoc correction; *p < 0.05; **p < 0.01, ***p < 0.001. Scale bar: 50 μm. **Figure S9.** EXO-depleted CM does not reverse hyperoxia-induced lung morphometric alterations **(A-D)** Histogram depicting number of branches **(A)**, junctions **(B)**, triple points **(C)**, quadruple points **(D)** in lungs of RA, BPD, DMEM:F12-, MSC-CM 25 wks- and EXO-depleted MSC-CM 25 wks-injected BPD mice at PN14. All values are expressed as mean ± SEM; five experiments, N = 3*–*5 mice per group; one-way ANOVA with Tukey’s post hoc correction; *p < 0.05; **p < 0.01. Scale bar: 50 μm. **Figure S10.** Elevated levels of tumor necrosis factor-α-stimulated gene-6 (TSG-6) in human BPD tracheal aspirate and lung tissue of the BPD mouse model and its detection in EXO fraction **(A)** Relative TSG-6 expression in the human lung tracheal aspirates (TA) from premature infants. Real-time PCR demonstrates elevated levels of TSG-6 in the BPD samples compared to the non-BPD samples. **(B)** Western blot quantitation of TSG-6 expression in RA, BPD, BPD + PBS, BPD + MSC-CM EXO 25 wks, BPD + MSC-CM EXO 30 wks groups at PN14. TSG-6 (~ 37 kDa) was detected by western blotting, and β-actin was used as control for quantitation of TSG-6 expression by densitometry. **(C)** Western blotting for detection of TSG-6 in MSC-CM and MSC-CM EXO from 25 wks GA UC. PBS and DMEM:F12 were loaded as controls. TSG-6 is expressed in both CM and EXO from 25 wks GA UC. No signal was detected in control samples used for vehicle injections, DMEM:F12 and PBS. β-actin was used as a control to depict the cytoskeletal content in different fractions. All values are expressed as mean ± SEM; N = 3–6 mice per group; one-way ANOVA with Tukey’s post hoc correction; *p < 0.05; **p < 0.01. **Figure S11**. TSG-6 treatment reverses morphometric alterations, decreases cell death, and rescues hyperoxia-induced loss of peripheral pulmonary blood vessels in the BPD mouse model. **(A-B)** Histogram depicting number of triple points **(A)** and quadruple points **(B)** in lungs of RA, BPD, PBS-injected and TSG-6 treated BPD mice at PN14. (**C)** Representative images of lung sections stained with endothelial cell marker CD31. *Arrows* point to CD31 staining (*green*) in the blood vessels in the lungs of RA (I), BPD (II), PBS-injected (III) and TSG-6-treated (IV) BPD mice at PN14 mice. The cells were counterstained with DAPI for nuclear staining. **(D)** Quantitative analyses of mean pixel intensity of CD31 immunofluorescence at PN14 in RA, BPD, PBS-injected and TSG-6-injected BPD mice groups. Scale bar: 100 μm. All values are expressed as mean ± SEM; four experiments, N = 3–8 mice per group; one-way ANOVA with Tukey’s post hoc correction; *p < 0.05; **p < 0.01; ***p < 0.001; ****p < 0.0001. **Figure S12**. TSG-6 treatment decreases hyperoxia-induced cell death and reversed loss of myelination and increased GFAP expression in the brain of BPD mice **(A-D).** Histogram demonstrating the quantitative analysis of TUNEL-positive cells, expressed as a percentage (as described in Additional Information methods) in the olfactory bulb region **(A)**, forebrain region **(B)**, midbrain region **(C)**, and hindbrain region **(D)** in RA, BPD, BPD+ PBS and BPD + TSG-6-injected PN14 mice brain. **(E-H)** TSG-6 administration attenuates demyelination and decrease in astrocytes in hyperoxia-induced lung injury **(E)** Representative immunofluorescence images of MBP staining in RA, BPD, vehicle (PBS)-injected BPD mice and TSG-6-injected BPD mice (I-IV) **(F)** Representative immunofluorescence images of GFAP staining in RA, BPD, PBS + BPD and TSG-6-injected groups (I-IV). ×400 magnification, Scale bar: 50 μm **(G)** Histogram showing quantitation of MBP expression in RA, BPD, BPD + PBS and TSG-6 groups. **(H)** Histogram showing quantitation of GFAP expression in RA, BPD, BPD + PBS and TSG-6 groups. All values are expressed as mean ± SEM; four experiments, N = 3–8 mice per group; one-way ANOVA with Tukey’s post hoc correction; *p < 0.05; **p < 0.01. **Figure S13. (A)** Histogram depicting the percentage of macrophages in the BALF of RA, BPD, PBS-injected, MSC-CM EXO 25 wks injected-, Isotype IgG+ EXO 25 wks injected-, NAb TSG-6 + EXO 25 wks injected- BPD mice at PN14 **(B)** Graph demonstrating PAAT values from the echocardiography of six experimental groups RA, BPD, PBS-injected, MSC-CM EXO 25 wks injected-, Isotype IgG+ EXO 25 wks injected-, NAb TSG-6 + EXO 25 wks injected-BPD mice at PN14. **(C-D)** Relative TSG-6 expression in 25 wks GA MSCs at different time points after transfection with scr siRNA and TSG-6 siRNA, at the concentrations of 5 nM **(C)** and 10 nM **(D)**. Real-time PCR was done to analyze the percentage knockdown of TSG-6 after incubation with siRNA for 6 h, 16 h and 24 h. **(E)** Histogram depicting the percentage of macrophages in the BALF of RA, BPD, PBS injected-, MSC-CM EXO 25 wks injected-, scr siRNA EXO 25 wks injected-, TSG-6 siRNA EXO 25 wks injected-BPD mice at PN14. **(F)** Graph demonstrating PAAT values from the echocardiography of six experimental groups RA, BPD, PBS injected-, MSC-CM EXO 25 wks injected-, scr siRNA EXO 25 wks injected-, TSG-6 siRNA EXO 25 wks injected-BPD mice at PN14. All values are expressed as mean ± standard error of the mean (SEM); six experiments, N = 3–6 mice per group; one-way ANOVA with Tukey’s post hoc correction; *p < 0.05; **p < 0.01; ***p < 0.001; ****p < 0.0001. (PDF 1628 kb)

## Abbreviations

ANOVA: One-way analysis of variance
BALF: Bronchoalveolar lavage fluid
BPD: Bronchopulmonary dysplasia
CM: Conditioned medium
DAPI: 4,6-Diamidino-2-phenylindole
ELISA: Enzyme-linked immunosorbant assay
EM: Electron microscopy
EV: Extracellular vesicle
EXO: Exosomes
FACS: Fluorescence-activated cell sorting
FBS: Fetal bovine serum
GA: Gestational age
GFAP: Glial fibrillary acidic protein
H&E: Hematoxylin and eosin
HDF: Human dermal fibroblast
hUC: Human umbilical cord
i.p.: Intraperitoneal
IL-1β: Interleukin-1 beta
IL-6: Interleukin-6
IVS: Interventricular septa
LV: Left ventricular
MBP: Myelin basic protein
MSC: Mesenchymal stem cell
NAb: Neutralizing antibody
NB: Newborn
NTA: Nanoparticle tracking analysis
PAAT: Pulmonary artery acceleration time
PAET: Pulmonary artery ejection time
PBS: Phosphate-buffered saline
PCR: Polymerase chain reaction
PFA: Paraformaldehyde
PH: Pulmonary hypertension
PN: Postnatal
RA: Room air
RV: Right ventricular
RVH: Right ventricular hypertrophy
scr siRNA: Scrambled small interfering ribonucleic acid
SFM: Serum-free media
TA: Tracheal aspirate
TEM: Transmission electron microscopy
TNF-α: Tumor necrosis factor alpha
TSG-6: Tumor necrosis factor alpha-stimulated gene-6
*TSG-6* siRNA: Tumor necrosis factor α stimulated gene-6 small interfering ribonucleic acid
TUNEL: Terminal deoxynucleotidyl transferase dUTP nick-end labeling
UC: Umbilical cord
WJ: Wharton’s jelly
Wks: Weeks
WT: Wildtype
