# Supplementary material for: Early gestational mesenchymal stem cell secretome attenuates experimental bronchopulmonary dysplasia in part via exosome-associated factor TSG-6
Source: Stem Cell Res Ther. 2018 Jun 26;9:173. doi: 10.1186/s13287-018-0903-4 (PMC6019224; doi:10.1186/s13287-018-0903-4)

Early Gestational Mesenchymal Stromal Cell Secretome attenuates Experimental Bronchopulmonary Dysplasia in part via Exosome-Associated Factor TSG-6

Sushma Chaubey, PhD[1], Sam Thueson, BS[1], Devasena Ponnalagu, PhD[2], Mohammad Afaque Alam, PhD[1], Ciprian P. Gheorghe, MD, PhD[3], Zubair Aghai, MD[4], Harpreet Singh, PhD[2,5], and Vineet Bhandari, MD, DM[1*]

[1]Section of Neonatal-Perinatal Medicine

Department of Pediatrics

Drexel University College of Medicine

245 N 15th Street, Philadelphia, PA 19102

Telephone (215) 762-7595 / Fax (215) 762-7960

[2]Department of Pharmacology & Physiology

[5]Department of Medicine, Division of Cardiology

Drexel University College of Medicine

245 N 15th Street, Philadelphia, PA 19102

Telephone (215) 762-4751 / Fax (215) 762-2299

[3]Department of Obstetrics and Gynecology

Loma Linda University School of Medicine

11370 Anderson Street, Loma Linda, CA 92354

[4]Divison of Neonatology, Department of Pediatrics

Thomas Jefferson University Hospital,

132S, 10th Street, Philadelphia, PA, USA 19107

*Corresponding Author: vineet.bhandari@drexel.edu

SUPPLEMENTARY INFORMATION

SUPPLEMENTARY EXPERIMENTAL PROCEDURES

Isolation and expansion of human WJ MSCs

UC-MSCs were isolated from preterm neonates of 25 and 30 wks GA UC WJ, using a modified protocol described previously(1). Briefly, the UCs were washed with cold Phosphate Buffer Saline (PBS) followed by short 70% (v/v) alcohol rinse and PBS wash. It was then cut into 1.5 cm length pieces, which were cut open lengthwise and UC vessels were removed. The gelatinous tissue surrounding the vessels was washed thoroughly with cold PBS until clean of cord blood and blood clots. Pieces were finely minced and digested in 0.1% (w/v) collagenase A (Sigma) in PBS at 37°C for 2h followed by DNAse I digestion for 1h. The suspension was then diluted 1:5 with PBS at room temperature and filtered through 70 µm sterile nylon cell strainers and centrifuged at 800 x g for 10 min at 4° C. The cell pellet was plated after resuspending in MSC culture media consisting of DMEM:F12 supplemented with 10% (v/v) FBS and 1% (v/v) Penicillin/Streptomycin/Fungizone. The MSC cultures were maintained at 37°C in humidified 5% carbon dioxide tissue culture incubators. Non-adherent cells and debris were removed after 48h. Adherent cells were fed every 2-3 days by changing media until cells formed a confluent monolayer. The cells were then passaged with 0.25% trypsin and subcultured. Passage 3 cells were used in all experiments. Mean population doubling time (Td) was calculated using the formula: Td = (t2-t1) x log 2/log (q2/q1) where q_1 at time t_1 and q_2 at time t_2, are two measurements of a growing quantity, assuming a constant growth rate.

Characterization of UC-MSCs by Fluorescence activated cells sorting (FACS) and differentiation into adipocytes, osteocytes and chondrocytes

Standard flow cytometry was performed to determine the presence of MSC markers which are defined by the International Society for Cellular Therapy (2). MSCs when confluent were resuspended in cold PBS and stained with viability dye eFluor 450 (Biosciences) for 20 min on ice. The cells were washed with cold PBS followed by staining with mouse monoclonal antibodies (mAb) from BioLegend: APC-conjugated anti-human CD105; Alexa Fluor 700 anti-human CD90 (Thy1) and phycoerythrin (PE)-conjugated anti-human CD73, to identify positive markers for MSCs. Alternatively, UC-MSCs were stained with Alexa Fluor 700 anti-human HLA-DR Antibody, PE/Cy7 anti-human CD14, PE-conjugated CD19. These antibodies were used to identify negative markers for MSCs. All incubations were done on ice to prevent antigen internalization. The cells were fixed in 1% paraformaldehyde before flow cytometric analysis. Data was collected on a FACS Fortessa using FACS Diva software (BD Biosciences). Analysis was performed using Flow Jo software (Tree Star).

UC-MSCs were differentiated into adipocytes, osteocytes and chondrocytes in vitro in appropriate differentiation media (StemPro Differentiation Kit, Gibco by Life Technologies), as per the manufacturer's instructions. Briefly, at 70-80% confluency, UC-MSCs were incubated in pre-warmed complete differentiation media with supplements. The differentiation media was changed every 3-4 days. After 21 days under differentiation conditions, the cells were washed with 1x PBS and fixed with 4% paraformaldehyde for 30 min at RT. After rinsing the cells with 1x PBS, the cells were stained with Oil Red O (for adipocytes), Alizarin Red S solution (for osteocytes) and alcian blue (for chondrocytes) as per the manufacturer's guidelines.

Bronchoalveolar lavage (BAL)

Mouse pups were euthanized by pentobarbital overdose (250 mg/kg intraperitoneal) at PN14. The trachea was exposed by blunt dissection, and a 24GA 0.56in caliber tube (BD Insyte-N TM Autogaurd shielded IV catheter) was inserted into the airway and secured. Three washes of 0.2 ml PBS were instilled and gently aspirated and pooled to obtain BAL fluid (BALF). A small volume of BALF was used to calculate total cell counts using a hemocytometer. BALF samples were then cytocentrifuged at 1500 g for 15 min at 4° C to recover cells and the supernatant were collected and stored at -80° C to estimate total BALF protein concentration using BCA kit (Pierce, Rockford, IL), as per the manufacturer's recommendations. Differential cell counts were obtained after staining with HEMA stain as previously described (4). Absolute neutrophil count was determined by counting a minimum of 300 cells in each sample and multiplying it with its total cell number. Percentage macrophage was calculated by examining the HEMA stained cells under microscope at 200x magnification. Five to six non-overlapping fields were examined to count a minimum of 300 cells. Percentage macrophages were expressed as (total number of macrophages/total number of cells) x100.

Western Blot analysis

The tissue was homogenized in RIPA buffer and protease inhibitor cocktail (Santa Cruz; USA) using tissue disrupter homogenizer. After centrifugation, the supernatant was lysed and subjected to sodium dodecyl sulfate polyacrylamide gel electrophoresis (SDS-PAGE). Samples equivalent to 30 µg of protein content was loaded and size separated by 4% -20% gradient SDS-PAGE (BioRad). The protein on the acrylamide gel were transferred to a polyvinylidene difluoride

(PVDF) membrane (Millipore, Bedford, MA, USA) or nitrocellulose membrane at constant voltage 90 V in a transfer buffer containing 25 mM Tris and 192 mM glycine, pH 8.4. The PVDF membrane was blocked in PBS with 5% (w/v) skimmed milk overnight followed by incubation with the appropriate primary antibodies TSG-6 (R&D systems, 1:800) overnight at 4°C. The membrane was washed thoroughly with PBS with 0.1% (v/v) Tween, five washes for 5 minutes each. The membrane was then incubated with horseradish peroxidase conjugated secondary antibody (anti-mouse IgG2B, Santa Cruz Inc.) and β-actin HRP-conjugate (Cell Signaling Technology, 1:3000) or secondary antibodies labeled with IRDye near-infrared fluorescent dyes (LI-COR Biosciences, 1:10,000). for 1h at room temperature. After PBS-Tween wash, the membranes were visualized by a chemiluminescent ECL detection kit from Perkin Elmer Inc. or by Odyssey Imaging system (LI-COR). For CD81 and Alix-1 staining, the blots were incubated with primary antibody mouse CD81 (Santa Cruz Inc. 1:200) or mouse Alix-1 (Santa Cruz Inc; 1:200) followed by incubation with anti-mouse LI-COR fluorescent secondary antibody. After PBS wash, the blots were scanned at appropriate wavelength using Odyssey Imaging system.

Dot Blot Assay

CM, EXO and their respective control fractions were spotted on the methanol-treated PVDF membranes and allowed to air dry at least for 2 h. The blots were rinsed briefly in PBS and were incubated overnight at 4° C in 5% (w/v) skimmed milk in PBS to block the residual binding sites in the membrane. The blots were washed three times in PBS-Tween (0.1% v/v Tween 20) for 10 min and were then incubated with mouse primary antibodies diluted in 3% (w/v) skimmed milk in PBS (anti-CD63, Santa Cruz: 1:200 dilution; anti-TGN48, Santa Cruz: 1:200) for 2h at 37° C. The blots were washed in PBS-Tween (0.1%) followed by appropriate secondary antibody incubation

at 37° C for 1 h 15 min. After washing with PBS-Tween, the blots were developed using Chemiluminescent ECL detection kit. A clearly defined black spot at the site where the antigen was spotted was considered a positive result. A trace reaction or absence of any reaction was considered a negative result.

Terminal Deoxynucleotidyl Transferase dUTP Nick End Labeling (TUNEL) assay

The immunofluorescent TUNEL staining with an *in situ* cell death detection kit (Fluorescein Version 17, Roche) was done to measure the extent of apoptosis in the lung and brain. Paraffin sections slides were de-paraffinized, rehydrated followed by antigen retrieval by heating it in microwave in citrate buffer pH 6.0. Sections were then washed with PBS and incubated in blocking solution (10% BSA with 0.3% Triton-X 100 in 1x PBS) for 30 min at 25° C. The slides were washed with 1x PBS for five times and then incubated with TUNEL reaction mixture (1 part of enzyme solution and 9 parts of label solution) for 1h at 37° C in dark. Slides were washed with PBS, mounted with Vectashield mounting solution with DAPI (Vector Laboratories) and visualized with a fluorescent microscope using an exciting wavelength of 460-490 nm. Eight to ten non-overlapping fields under 400x magnification was examined to count TUNEL positive cells. Percentage TUNEL positive cells were expressed as (total number of green positive cells/total number of DAPI positive cells) x100.

Immunofluorescence and Immunohistochemistry

Immunofluorescence and immunohistochemistry was performed on de-paraffinized 5 µm thick lung section embedded tissue sections. Antigen retrieval was performed for all slides with 10 mM citrate buffer pH 6.0 (Sigma). Primary antibodies incubations were performed overnight at 4° C.

The primary antibodies were used in following concentrations: cleaved caspase-3 (Cell signaling, 1:300), CD31 (BD Biosciences 1:200), MBP (Santa Cruz Inc. 1:200), GFAP (Millipore MAB3402 clone GA5, 1:300). Secondary antibodies used were from Jackson Immunoresearch, 1:300 dilutions. Sections were mounted with Vectashield mounting medium containing DAPI according to manufacturer's instructions (Vector Laboratories, California). The stained sections were visualized using immunofluorescence microscope. Six-eight images per sample were captured at 400x magnification for quantification of cleaved caspase-3, MBP and GFAP, and 100x magnification for quantification of CD31 expressing cells. Quantification was done using ImageJ software (NIH).

Enzyme-Linked Immunosorbant Assay (ELISA)

Protein was isolated from the lungs of RA, BPD and MSC-CM/EXO/TSG-6 treated BPD mice. Total protein content was measured by BCA kit (BioRad). Concentration of mouse IL-6 in the lung homogenates was analyzed using ELISA kit (DuoSet IL-6, R&D Systems) as per manufacturer's instructions

Supplementary Information Table S1. Comparison of different parameters in the exosome dose.

**Parameters**	**hUC WJ derived MSC-CM EXO 25 wks**	**hUC WJ derived MSC-CM EXO 30 wks**	**WJMSC-EXO (Willis et al. 2017)**	**BMSC-EXO (Willis et al. 2017)**	**HDF-EXO (Willis et al. 2017)**
**Volume injected**	100 µl	100 µl	50 µl	50 µl	50 µl
**Delivery route**	IP	IP	IV	IV	IV
**NTA (particles)**	$4.5x10^{8}$	2.88×10^{7}	8.5×10^{8}	7.2×10^{8}	9.6×10^{8}
**Protein**	2.8 µg	2.4 µg	0.9 µg	3 µg	1.5 µg
**Cell equivalents**	0.7 × 106	0.7 × 106	0.5 × 106	0.5 × 106	0.5 × 106
**Serum free media (SFM) exposure**	24 h	24 h	36 h	36 h	36 h

Supplementary Information Table S2. Clinical data for BPD and No BPD (n=10)

Parameters	All	BPD	No BPD
**Birth Weight (grams)**	761±206	642±116	881±251
**Gestational Age (weeks)**	25.8±2.0	24.6±1.4	27.0±2.2
**Female Sex (%)**	7 (70)	3 (60)	4 (80)
**White Race (%)**	4 (40)	1 (20)	3 (60)
**Prenatal steroids (%)**	10 (100)	5 (100)	5 (100)
**Surfactant (%)**	10 (100)	5 (100)	5 (100)
**Late onset sepsis (%)**	5 (50)	3 (60)	2 (40)
**Severe IVH (Grade 3 or more)**	6 (60)	4 (80)	2 (40)

No significant difference between BPD and No BPD (p=0.06 for Body weight and GA). IVH: Intraventricular hemorrhage

SUPPLEMENTARY REFERENCES

1. Mennan C, Wright K, Bhattacharjee A, Balain B, Richardson J, Roberts S. Isolation and characterisation of mesenchymal stem cells from different regions of the human umbilical cord. *BioMed research international* 2013; 2013: 916136.

2. Dominici M, Le Blanc K, Mueller I, Slaper-Cortenbach I, Marini F, Krause D, Deans R, Keating A, Prockop D, Horwitz E. Minimal criteria for defining multipotent mesenchymal stromal cells. The International Society for Cellular Therapy position statement. *Cytotherapy* 2006; 8: 315-317.

3. Bhandari V, Choo-Wing R, Homer RJ, Elias JA. Increased hyperoxia-induced mortality and acute lung injury in IL-13 null mice. *Journal of immunology* 2007; 178: 4993-5000.

4. Willis GR, Fernandez-Gonzalez A, Anastas J, Vitali SH, Liu X, Ericsson M, Kwong A, Mitsialis SA, Kourembanas S. Mesenchymal Stromal Cell Exosomes Ameliorate Experimental Bronchopulmonary Dysplasia and Restore Lung Function Through Macrophage Immunomodulation. *American journal of respiratory and critical care medicine* 2017.

SUPPLEMENTARY FIGURE LEGENDS

Supplementary Fig. S1. Preterm hUCs MSCs, but not fibroblasts, improve the BPD pulmonary phenotype. i.p. injections of MSCs from preterm (32 weeks) hUCs, in our mouse BPD model, at PN2 and PN4 showed improvement in lung architecture (at PN14), as evidenced by decreased chord length compared to the BPD mice. Human primary dermal fibroblast (HDF) cells, injected as control cells in the BPD mice, demonstrated no difference as compared to BPD

mice. All values are expressed as mean ± standard deviation (SD); 4 experiments, N=3 *mice per group;* one-way ANOVA with Tukey's post hoc correction; **p* <0.05; **p <0.01; ****p* <0.001.

Supplementary Fig. S2. Isolation and characterization of preterm UC-MSCs (A) UC-MSCs from 25 and 30 wks gestational age UC, after Passage 1, 2 days (I, III) and 5 days (II, IV) in culture respectively. The cells were adherent to the culture flasks and displayed fibroblast-like morphology. (B) FACS characterization of UC-MSCs. UC-MSCs were positive for CD105, CD73and CD90 and were negative for HLA-DR, CD19 and CD14. (C) Differentiation of UC-MSCs into adipocytes (Oil Red O staining), osteocytes (Alizarin Red S staining) and chondrocytes (Alcian Blue staining).

Supplementary Fig. S3. (A-F). Isolation and identification of hUC MSC-CM EXO (A) Transmission electron micrograph (TEM) of hUC-MSC-EXO 25 wks **(I)** and 30 wks **(II)** depicts intact exosomes with a diameter of 40-120 nm. Scale bar: 100 nm. **(B)** Particle number and size of isolated exosomes by nanosight nanoparticle tracking analysis. Representative plot of the 25wks EXO and 30 wks EXO, samples 1:50 and 1:20 diluted, respectively, for the analysis. **(C)** Dot blot of UC-MSC-CM 25 wks (I), UC-MSC-CM 25 wks EXO (II), UC-MSC-CM 30 wks (III), UC-MSC-CM EXO 30 wks (IV), show positive signal for exosome-specific antibody CD63 while no signal is detected in negative controls - DMEM:F12 media alone (V) or PBS (VI). **(D)** Dot blot of exosomal fractions [hUC-MSC-CM EXO 25 wks (II), hUC-MSC-CM EXO 30 wks (IV)] did not show any signal after probing with cytoskeletal membrane antibodies (TGN 48) which was otherwise detected in CM fractions [from 25 wks GA UC (I) and 30 wks GA UC (III)] confirming the purity of the isolated exosomal fraction. No signal was detected in negative controls -

DMEM:F12 (V) and PBS (VI). **(E)** Detection of CD81 in UC-MSCs, MSC-CM and MSC-CM EXO from 25 wks GA and 30 wks GA by Western blotting. Specific signal (25 kDa) was detected for CD81 in all fractions containing exosomes. No signal was seen in the loading controls, DMEM:F12 and PBS. Exosomal protein CD81 was also not detected in the EXO-depleted MSC-CM. **(F)** Detection of exosomal protein Alix-1 (97 kDa) in exosomes derived from 25 wks GA hUC MSCs by Western blotting.

Supplementary Fig. S4. (A) Percentage of macrophages in BALF. Histogram depicting the percentage of macrophages in the BALF of RA, BPD, DMEM:F12 or PBS-injected, MSC-CM or EXO (25 wks-injected, 30 wks) -injected BPD mice at PN14. **(B-C) Number of triple points and quadruple points.** Histogram depicting number of triple points **(B)** and quadruple points **(C)** in lungs of RA, BPD, DMEM:F12 or PBS-injected, MSC-CM or EXO (25 wks-injected, 30 wks) -injected BPD mice at PN14. **(D-E) Decreased hyperoxia-induced cell death in lungs of hUC MSC-CM treated BPD pups. (D)** Representative TUNEL immunofluorescence images of lung from the five experimental groups, RA (I), BPD (II), BPD + DMEM:F12 (III), BPD + MSC-CM 25 wks (IV), BPD + MSC-CM 30 wks (V). The nuclei are counterstained with DAPI. Arrows depict TUNEL-positive dead cells (green) in the field. Scale bar: 50 µm. **(E)** Histogram demonstrating the quantitative analysis of TUNEL positive cells, expressed as a percentage, as described in **Supporting Information** methods. All values are expressed as mean ± SEM; 5 experiments, N=3-5 *mice per group*; one-way ANOVA with Tukey's post hoc correction; *$p < 0.05$; **p <0.01; ***p <0.001; ****p <0.0001.

Supplementary Fig. S5. Decreased hyperoxia-induced cell death in lungs of hUC MSC-CM

EXO-treated BPD pups. (A) Representative TUNEL immunofluorescence images of lung from the five experimental groups, RA (I), BPD (II), BPD + PBS (III), BPD + MSC-CM EXO 25 wks (IV), BPD + MSC-CM EXO 30 wks (V). The nuclei are counterstained with DAPI. Arrows depict TUNEL-positive dead cells (green) in the field. Scale bar: 50 µm. **(B)** Histogram demonstrating the quantitative analysis of TUNEL positive cells, expressed as a percentage, as described in **Supplementary Information** methods. **(C)** Representative immunofluorescence images of lung from the five experimental groups, RA (I), BPD (II), BPD + PBS (III), BPD + MSC-CM EXO 25 wks (IV), BPD + MSC-CM EXO 30 wks (V) stained with cleaved caspase-3. The nuclei are counterstained with DAPI. Arrows depict TUNEL-positive dead cells (red) in the field. Scale bar: 50 µm. **(D)** Histogram demonstrating the quantitative analysis of cleaved caspase-3 positive cells, expressed as a percentage, as described in **Supplementary Information** methods. All values are expressed as mean ± SEM; 5 experiments, N=3-*5 mice per group*; one-way ANOVA with Tukey's post hoc correction; *$p < 0.05$; **p <0.01; ***p <0.001; ****p <0.0001.

Supplementary Fig. S6. MSC-CM EXO rescue hyperoxia-induced loss of peripheral pulmonary blood vessels (A) Representative images of lung sections stained with endothelial cell marker CD31. Arrows points CD31 staining (green) in the blood vessels in the lungs of PN14 mice. The cells were counterstained with DAPI for nuclear staining. **(B)** Quantitative analyses of mean pixel intensity of CD31 immunofluorescence at PN14 in RA, BPD, PBS-injected and MSC-CM-EXO-injected BPD mice groups. Scale bar: 100 µm. All values are expressed as mean ± SEM*;* 5 experiments, N=3-*5 mice per group*; one-way ANOVA with Tukey's post hoc correction; *p <0.05; **p <0.01. Scale bar: 50 µm.

Supplementary Fig. S7. hUC MSC secretome treatment decreases hyperoxia-induced cell death in the brain of the BPD mice. **(A-D)** Histogram demonstrating the quantitative analysis of TUNEL positive cells, expressed as a percentage in the olfactory bulb **(A)**, forebrain region **(B),** midbrain region **(C)** and hindbrain **(D)** region of PN14 mouse brain after MSC-CM treatment. **(E-H)** Histogram demonstrating the quantitative analysis of TUNEL positive cells, expressed as a percentage in the olfactory bulb **(E)**, forebrain region **(F),** midbrain region **(G)** and hindbrain **(H)** region of PN14 mouse brain after MSC-CM EXO treatment. All values are expressed as mean ± SEM*;* 5 experiments, N=*3-4 mice per group*; one-way ANOVA with Tukey's post hoc correction; **p* <0.05; **p <0.01; ****p* <0.001; ****p <0.0001.

Supplementary Fig. S8. hUC MSC-CM EXO treated pups decreased loss of myelination and increased GFAP expression in the brain of BPD mice. (A) Representative immunofluorescence images of MBP staining, marker for myelin, in RA, BPD, vehicle (PBS)-injected BPD mice, MSC-CM EXO 25 wks and MSC-CM EXO 30 wks-injected BPD mice (I, II, III, IV, V), to quantitate myelin loss in BPD and vehicle (PBS)-injected BPD mice (II, III) and restoration of myelination after MSC-CM EXO 25 wks (IV) and MSC-CM EXO 30 wks (V) injections. **(B)** Representative immunofluorescence images of GFAP staining in RA, BPD, PBS+BPD and MSC-CM EXO 25 wks and MSC-CM EXO 30 wks-injected BPD mice (I, II, III, IV, V). 400x magnification, Scale bar: 50 µm **(C-D)** Histogram showing quantitation of MBP expression **(C)** and GFAP expression **(D)** in RA, BPD, BPD+PBS, BPD+MSC-CM EXO 25 wks and BPD+MSC-CM EXO 30 wks groups. All values are expressed as mean ± SEM; 5 experiments, *N= 3-4 in each group*; one-way ANOVA with Tukey's post hoc correction; **p* <0.05; **p <0.01, ***p<0.001. Scale bar: 50 µm.

Supplementary Fig. S9. EXO-depleted CM does not reverse hyperoxia-induced lung morphometric alterations (A-D) Histogram depicting number of branches **(A)**, junctions **(B)**, triple points **(C)**, quadruple points **(D)** in lungs of RA, BPD, DMEM:F12-, MSC-CM 25 wks- and EXO-depleted MSC-CM 25 wks-injected BPD mice at PN14. All values are expressed as mean ± SEM*;* 5 experiments, N=3-*5 mice per group*; one-way ANOVA with Tukey's post hoc correction; **p* <0.05; **p <0.01. Scale bar: 50 µm.

Supplementary Fig. S10. Elevated levels of tumor necrosis factor α stimulated gene-6 (**TSG-6) in Human BPD tracheal aspirate and lung tissue of the BPD mouse model and its detection in EXO fraction (A)** Relative TSG-6 expression in the human lung tracheal aspirates (TA) from premature infants. Real-time PCR demonstrates elevated levels of TSG-6 in the BPD samples compare to the non-BPD samples. **(B)** Western blot quantitation of TSG-6 expression in RA, BPD, BPD+PBS, BPD+MSC-CM EXO 25 wks, BPD+MSC-CM EXO 30 wks groups at PN14. TSG-6 (~37 kDa) was detected by western blotting, and β-actin was used as controls for quantitation of TSG-6 expression by densitometry. **(C)** Western blotting for detection of TSG-6 in MSC-CM and MSC-CM EXO from 25 wks GA UC. PBS and DMEM:F12 were loaded as controls. TSG-6 is expressed in both CM and EXO from 25 wks GA UC. No signal was detected in control samples used for vehicle injections, DMEM:F12 and PBS. β-actin was used as a control to depict the cytoskeletal content in different fractions. All values are expressed as mean ± SEM*;* N=3-*6 mice per group*; one-way ANOVA with Tukey's post hoc correction; **p* <0.05; **p <0.01.

Supplementary Fig. S11. TSG-6 treatment reverses morphometric alterations, decreases cell death, and rescues hyperoxia-induced loss of peripheral pulmonary blood vessels in the BPD mouse model. (A-B) Histogram depicting number of triple points **(A)** and quadruple points **(B)** in lungs of RA, BPD, PBS-injected and TSG-6 treated BPD mice at PN14. (**C)** Representative images of lung sections stained with endothelial cell marker CD31. Arrows points CD31 staining (green) in the blood vessels in the lungs of RA (I), BPD (II), PBS-injected (III) and TSG-6 treated (IV) BPD mice at PN14 mice. The cells were counterstained with DAPI for nuclear staining. **(D)** Quantitative analyses of mean pixel intensity of CD31 immunofluorescence at PN14 in RA, BPD, PBS-injected and TSG-6-injected BPD mice groups. Scale bar: 100 µm. All values are expressed as mean ± SEM*;* 4 experiments, N=3-*8 mice per group*; one-way ANOVA with Tukey's post hoc correction; **p* <0.05; **p <0.01; ****p* <0.001; ****p <0.0001.

Supplementary Fig. S12. TSG-6 treatment decreases hyperoxia-induced cell death and reversed loss of myelination and increased GFAP expression in the brain of BPD mice (A-D) Histogram demonstrating the quantitative analysis of TUNEL positive cells, expressed as a percentage (as described in **Supplementary Information** methods) in the olfactory bulb region **(A)**, forebrain region **(B)**, midbrain region **(C)**, and hindbrain region **(D)** in RA, BPD, BPD+ PBS and BPD+TSG-6-injected PN14 mice brain. **(E-H)** TSG-6 administration attenuates demyelination and decrease in astrocytes in hyperoxia-induced lung injury **(E)** Representative immunofluorescence images of MBP staining in RA, BPD, vehicle (PBS)-injected BPD mice and TSG-6-injected BPD mice (I-IV) **(F)** Representative immunofluorescence images of GFAP staining in RA, BPD, PBS+BPD and TSG-6-injected groups (I-IV). 400x magnification, Scale bar: 50 µm **(G)** Histogram showing quantitation of MBP expression in RA, BPD, BPD+PBS and TSG-

6 groups. **(H)** Histogram showing quantitation of GFAP expression in RA, BPD, BPD+PBS and TSG-6 groups. All values are expressed as mean ± SEM; 4 experiments, N=3-*8 mice per group*; one-way ANOVA with Tukey's post hoc correction; *p < 0.05; **p <0.01.

Supplementary Fig. S13. (A) Histogram depicting the percentage of macrophages in the BALF of RA, BPD, PBS-injected, MSC-CM EXO 25 wks injected-, Isotype IgG+ EXO 25 wks injected-, NAb TSG-6+EXO 25 wks injected- BPD mice at PN14 **(B)** Graph demonstrating PAAT values from the echocardiography of six experimental groups RA, BPD, PBS-injected, MSC-CM EXO 25 wks injected-, Isotype IgG+ EXO 25 wks injected-, NAb TSG-6+EXO 25 wks injected- BPD mice at PN14. **(C-D)** Relative TSG-6 expression in 25 wks GA MSCs at different time points after transfection with scr siRNA and TSG-6 siRNA, at the concentrations of 5nM **(C)** and 10nM **(D)**. Real time PCR was done to analyze the percentage knockdown of TSG-6 after incubation with siRNA for 6h, 16h and 24h. **(E)** Histogram depicting the percentage of macrophages in the BALF of RA, BPD, PBS injected-, MSC-CM EXO 25 wks injected-, scr siRNA EXO 25 wks injected-, TSG-6 siRNA EXO 25 wks injected- BPD mice at PN14. **(F)** Graph demonstrating PAAT values from the echocardiography of six experimental groups RA, BPD, PBS injected-, MSC-CM EXO 25 wks injected-, scr siRNA EXO 25 wks injected-, TSG-6 siRNA EXO 25 wks injected- BPD mice at PN14. All values are expressed as mean ± standard error of the mean (SEM); 6 experiments, N=3-6 mice per group; one-way ANOVA with Tukey's post hoc correction; *p <0.05; **p <0.01; ***p <0.001; ****p <0.0001.

Supplementary Fig. S1

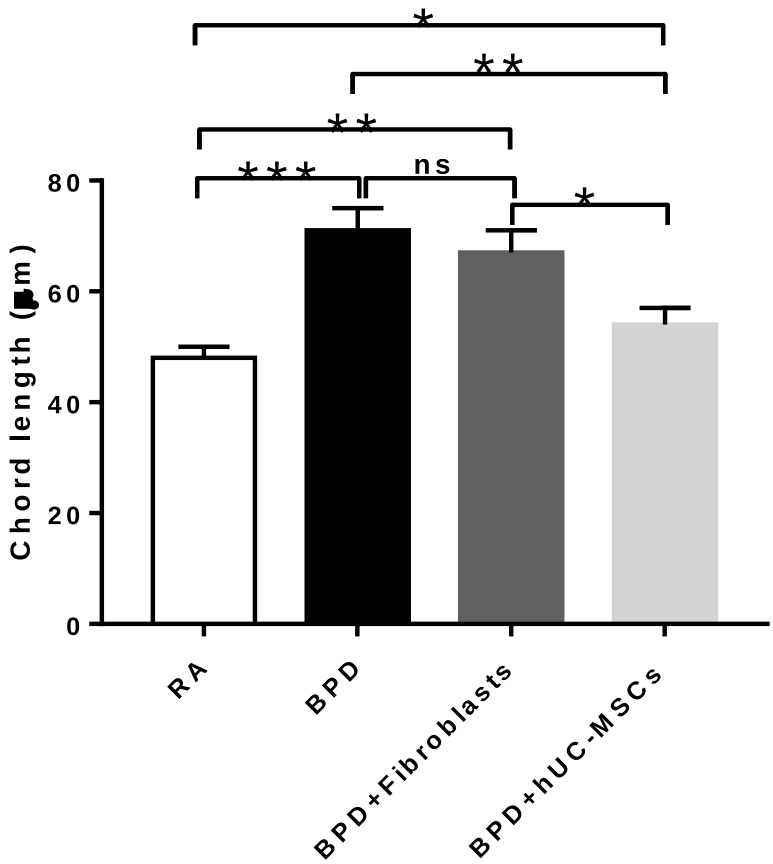

Supplementary Fig. S2

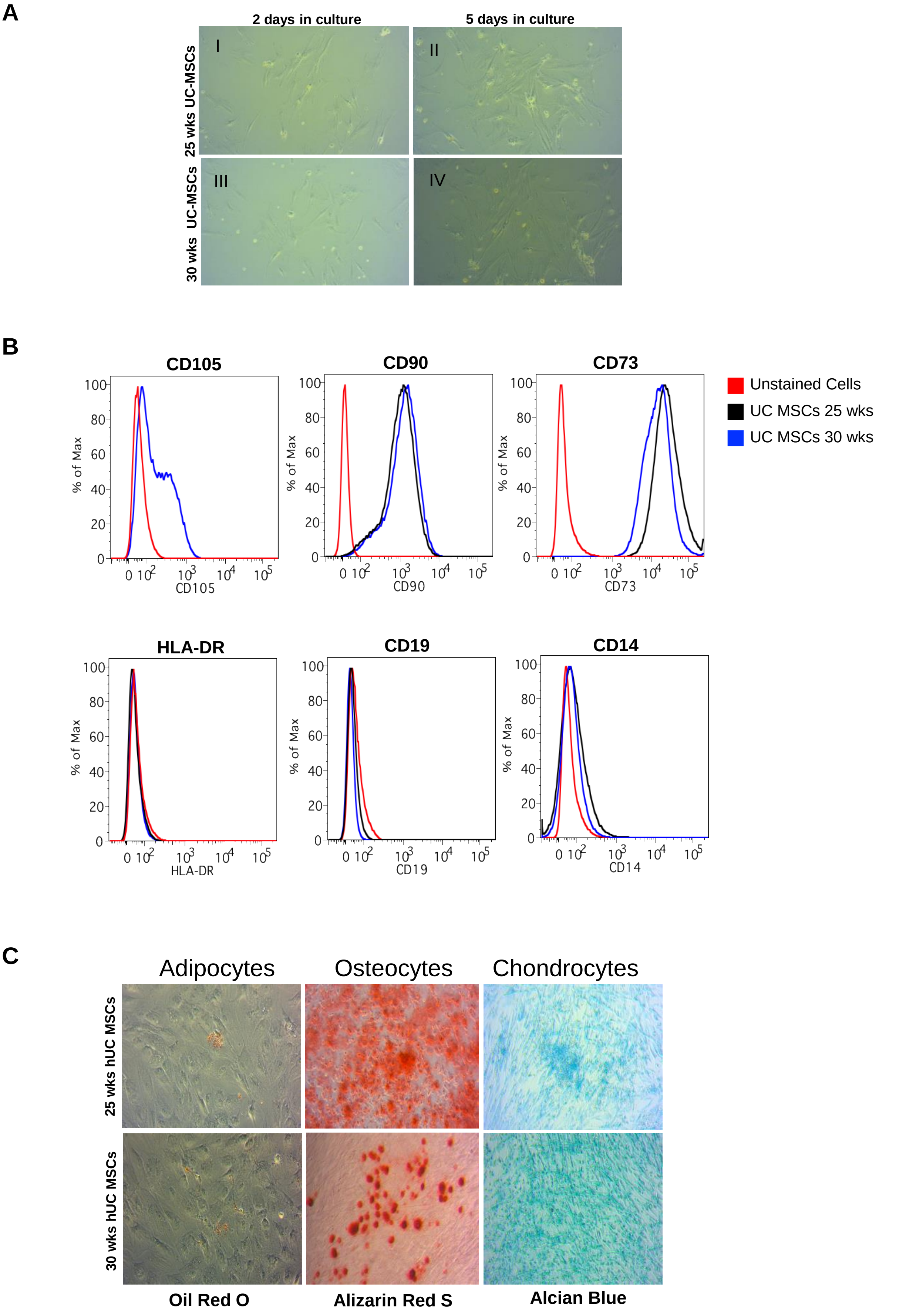

Supplementary Fig. S3

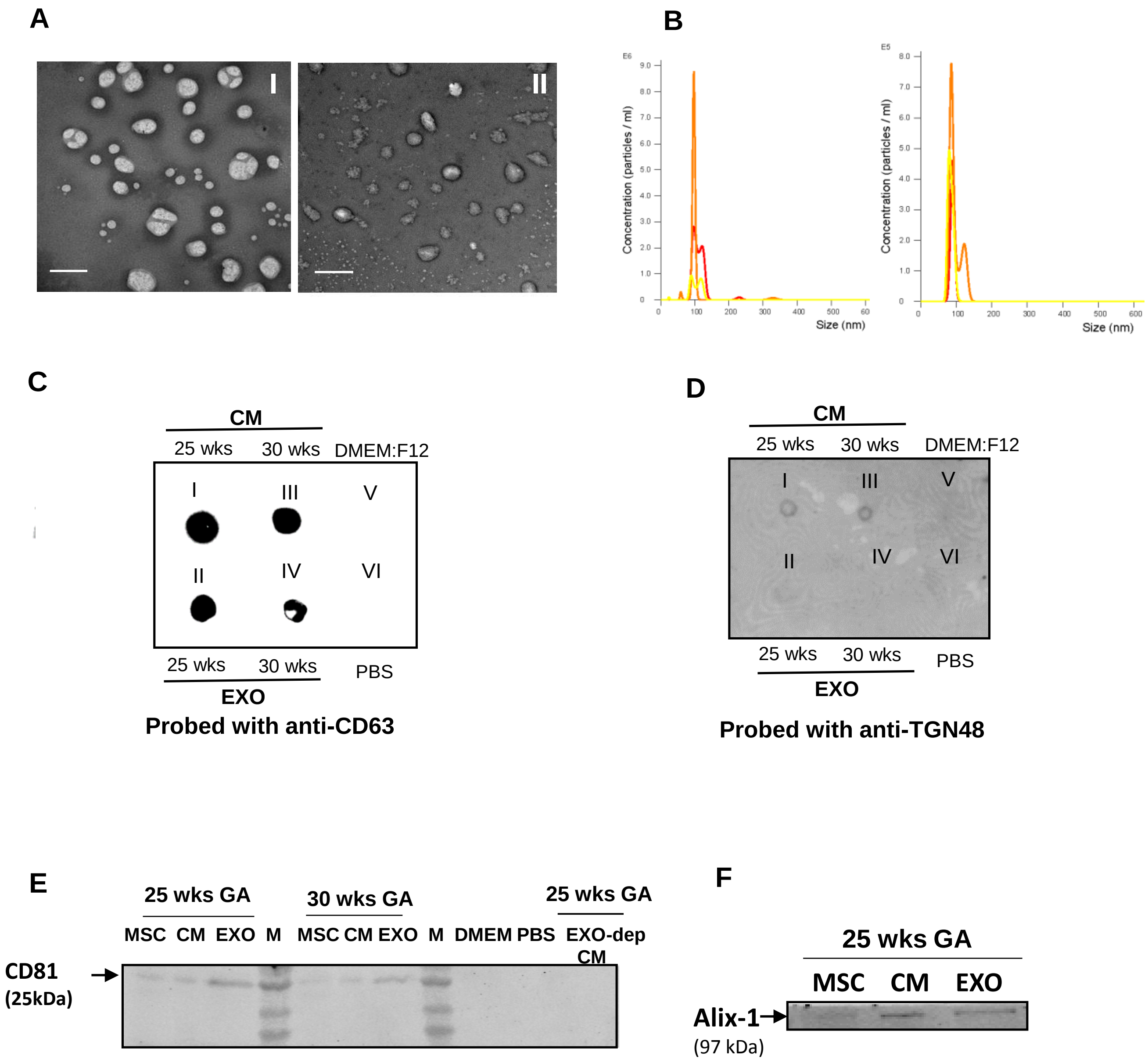

Supplementary Fig. S4

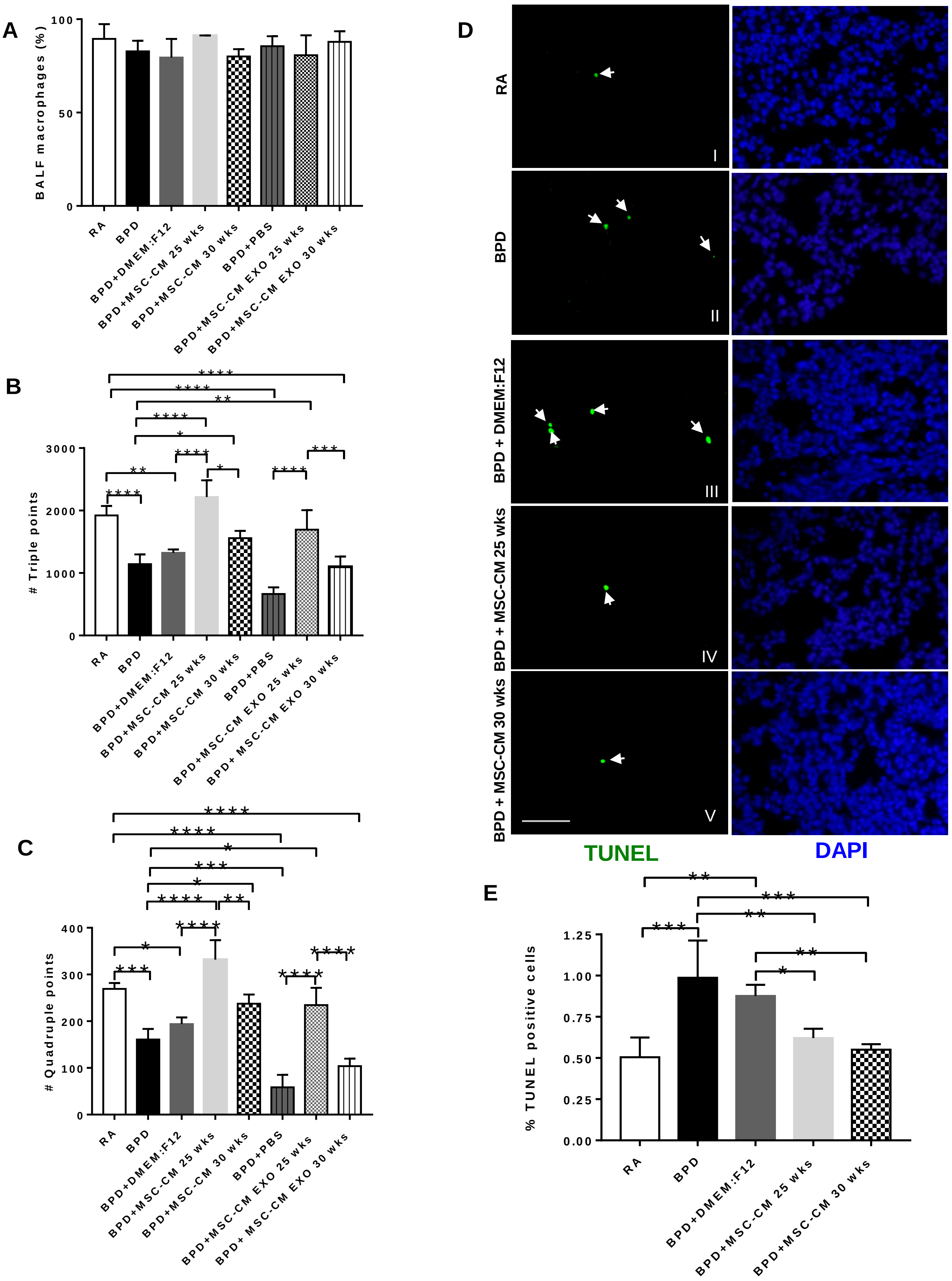

Supplementary Fig. S5

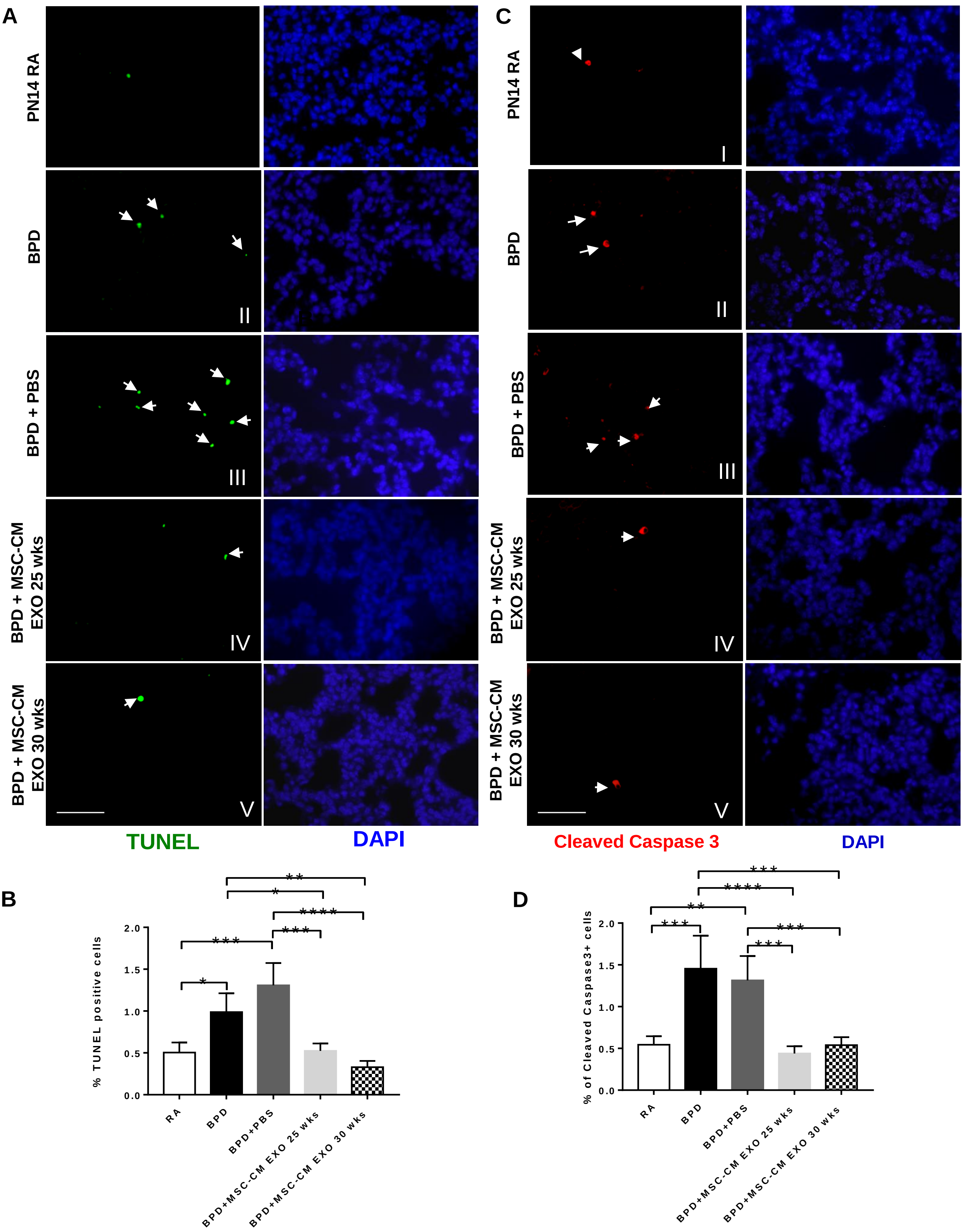

Supplementary Fig. S6

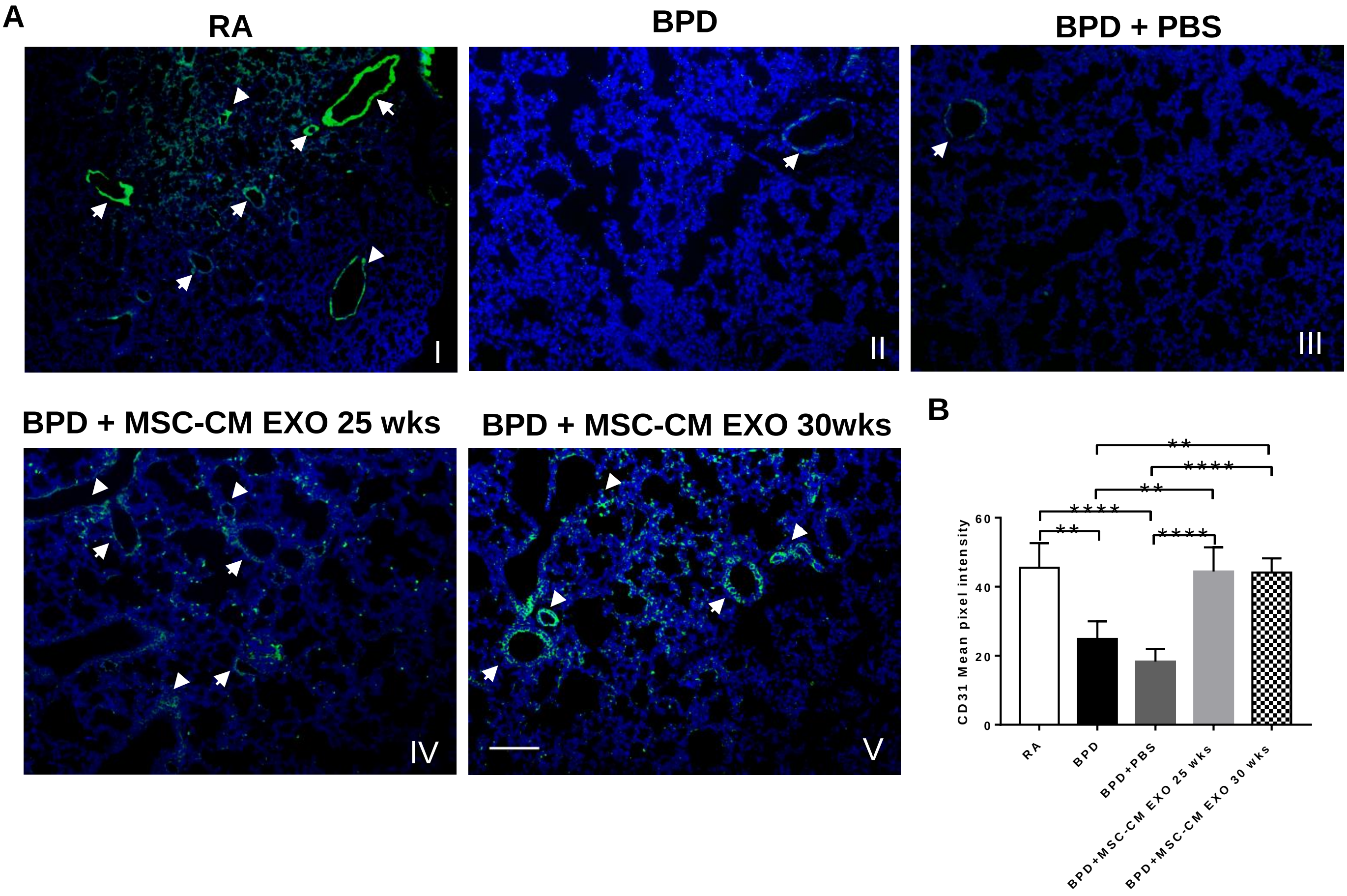

Supplementary Fig. S7

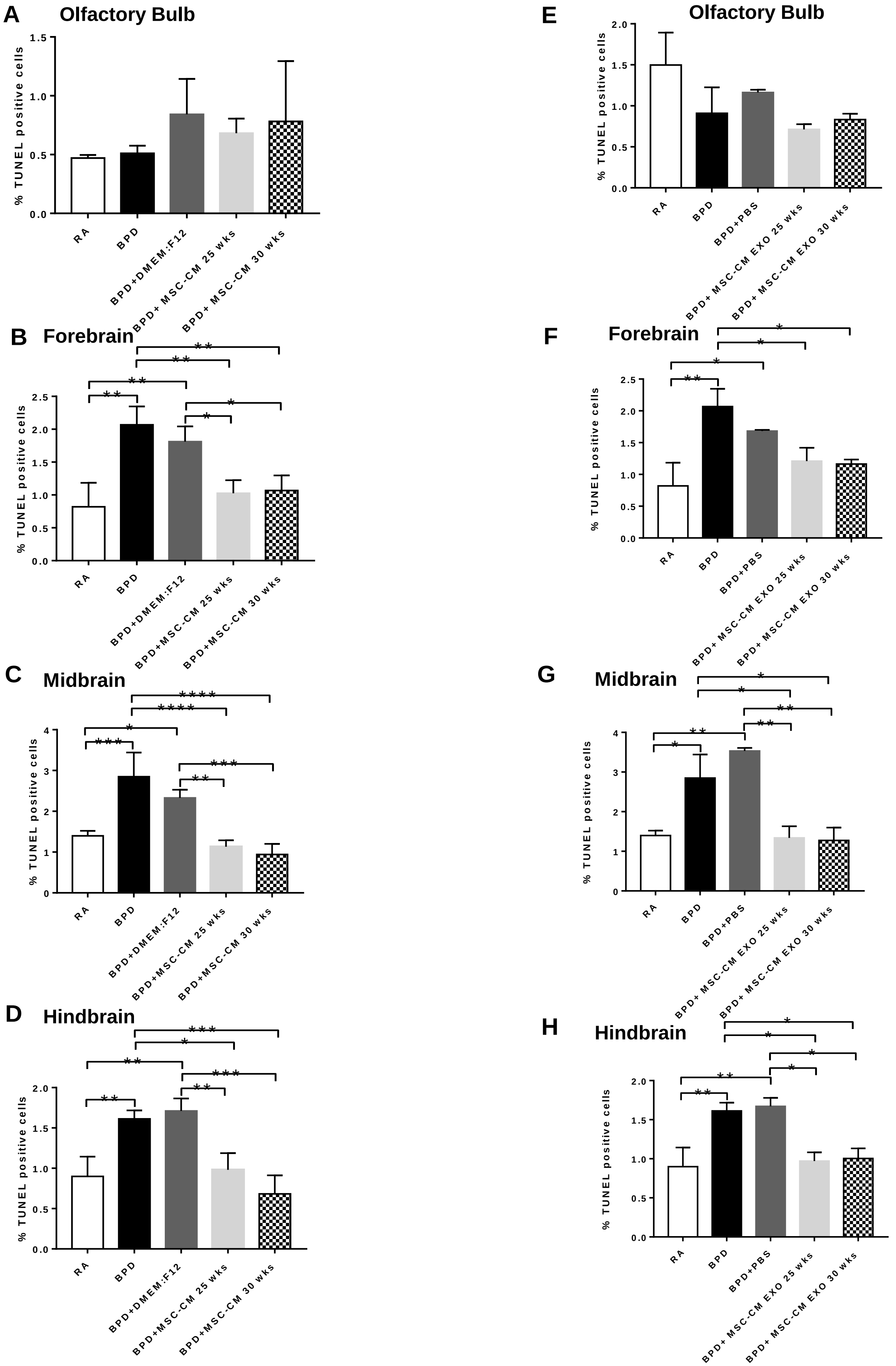

Supplementary Fig. S8

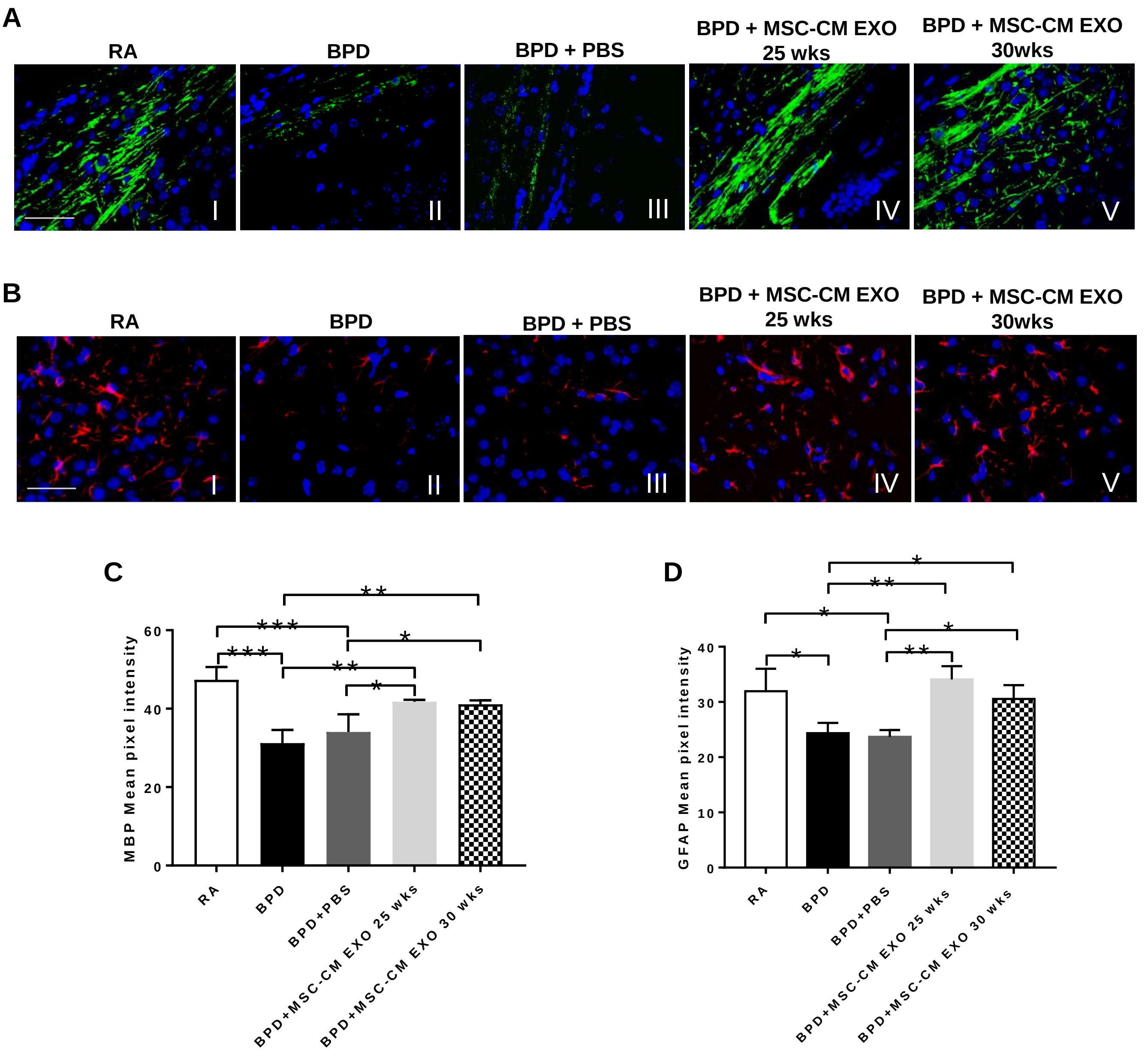

Supplementary Fig. S9

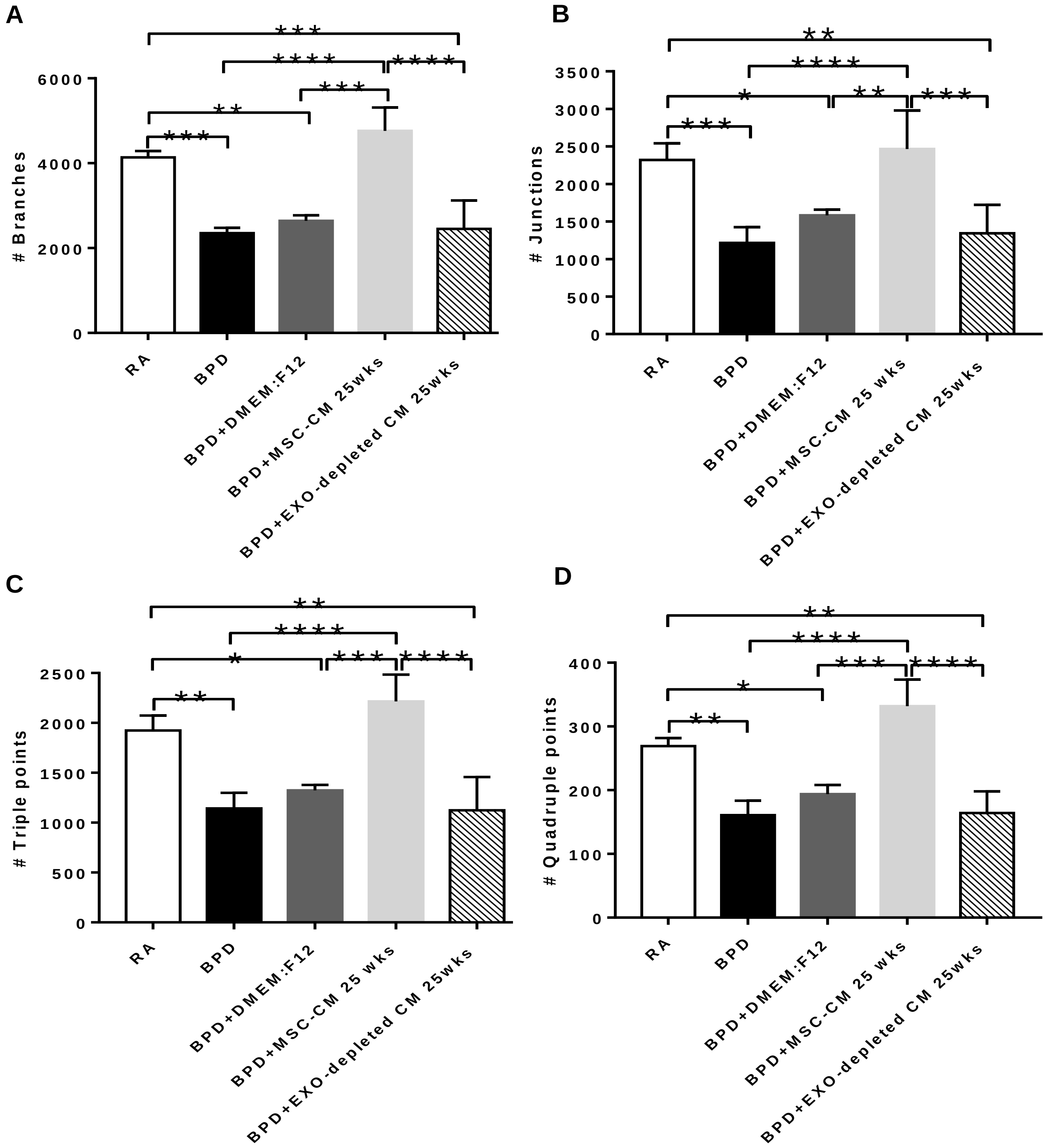

Supplementary Fig. S10

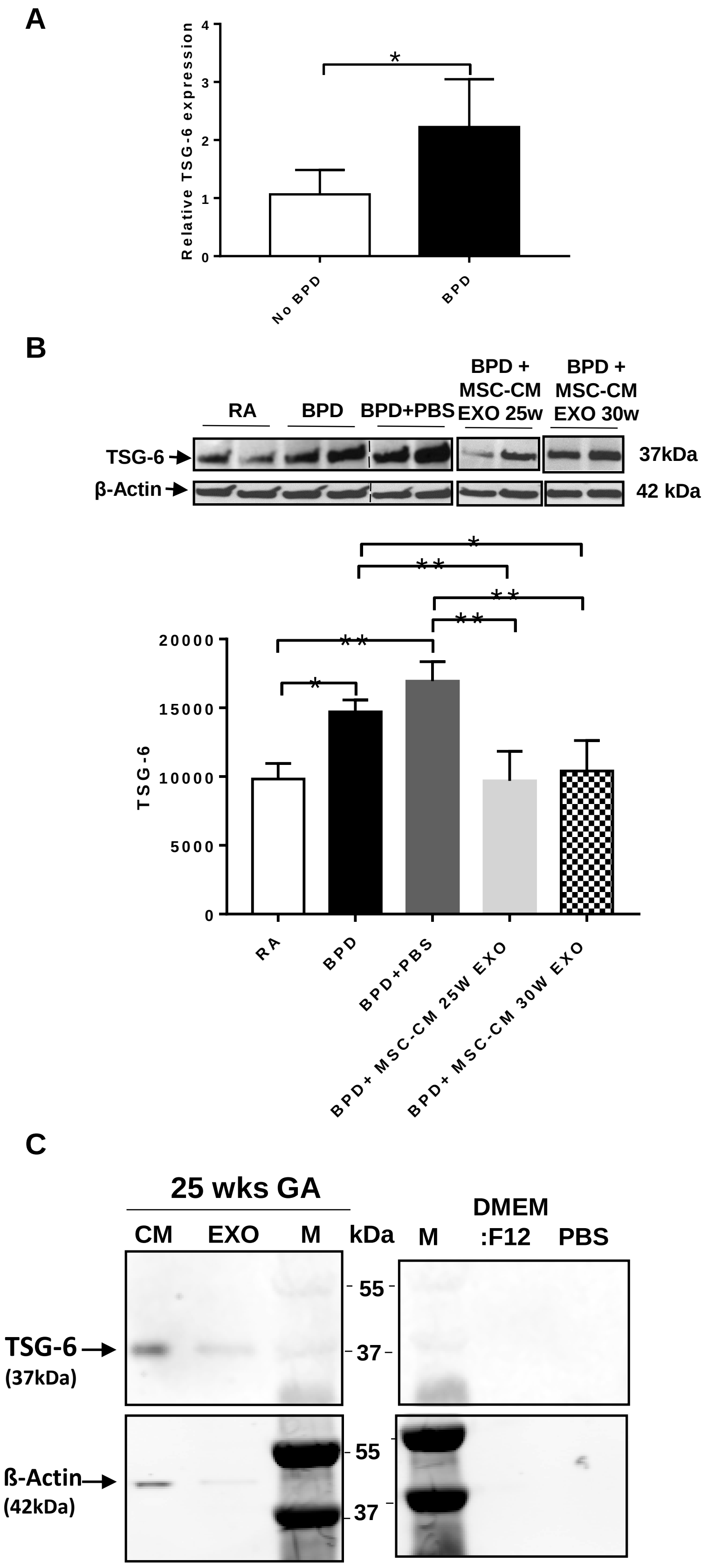

Supplementary Fig. S11

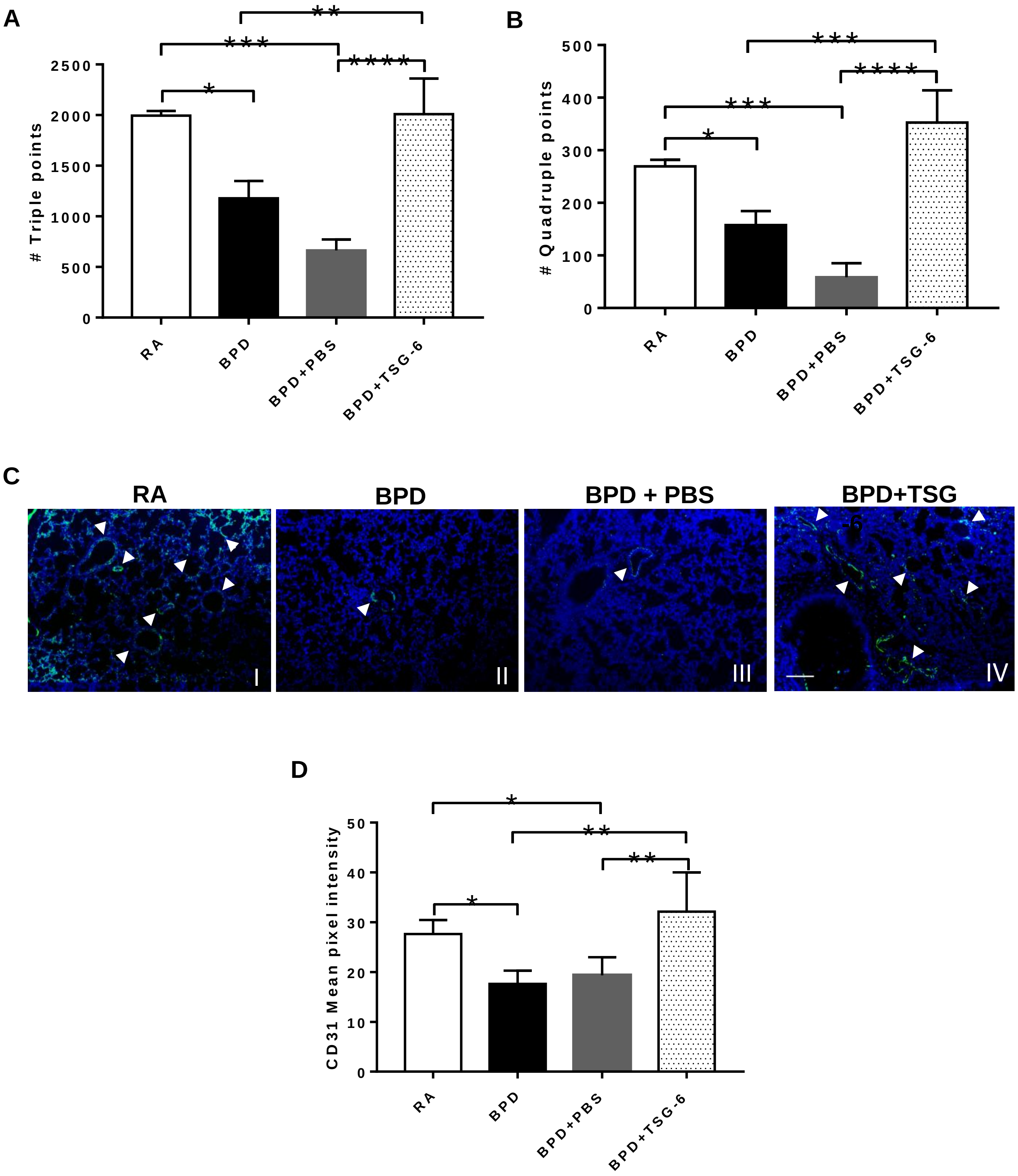

Supplementary Fig. S12

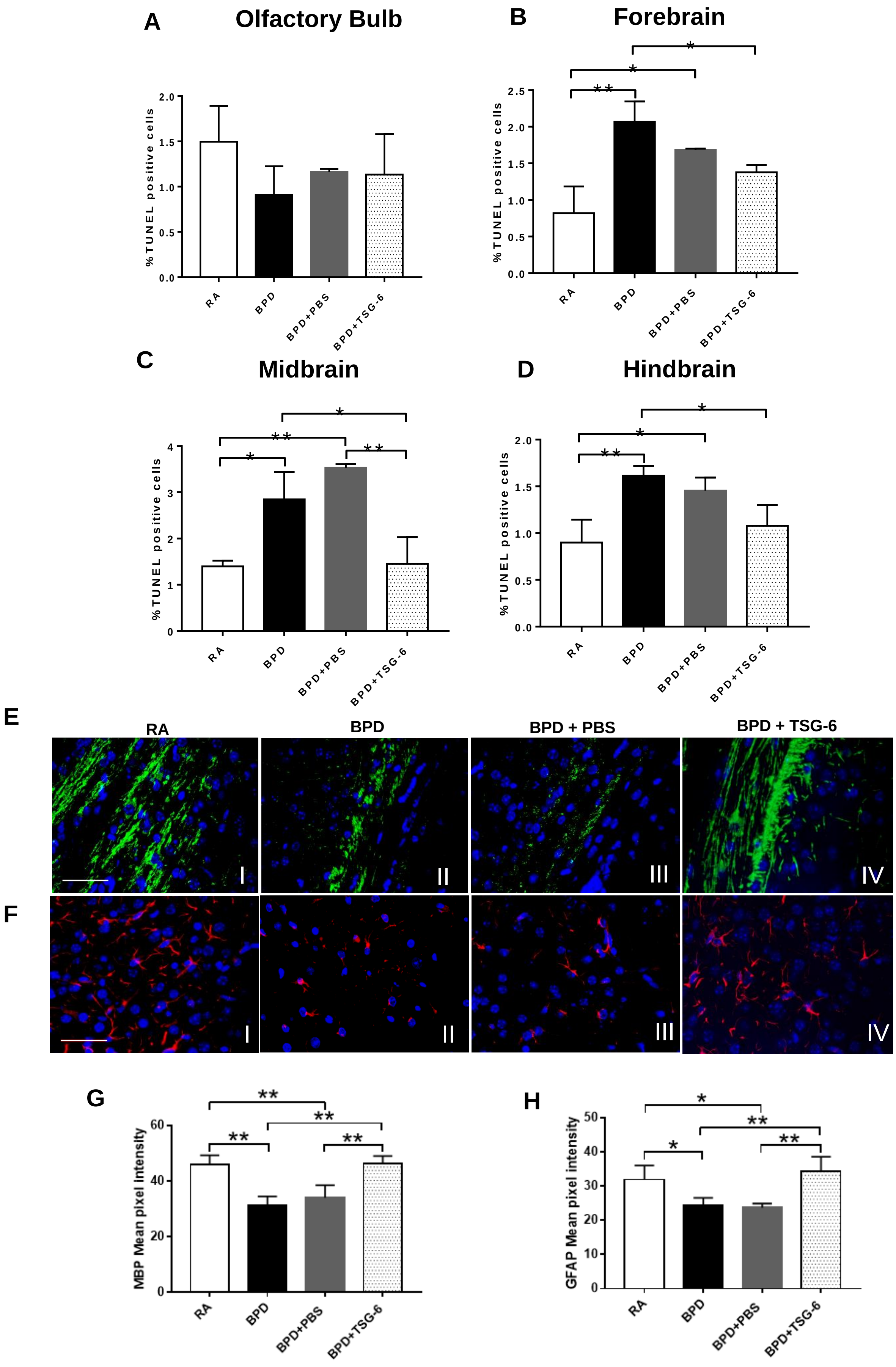

Supplementary Fig. S13

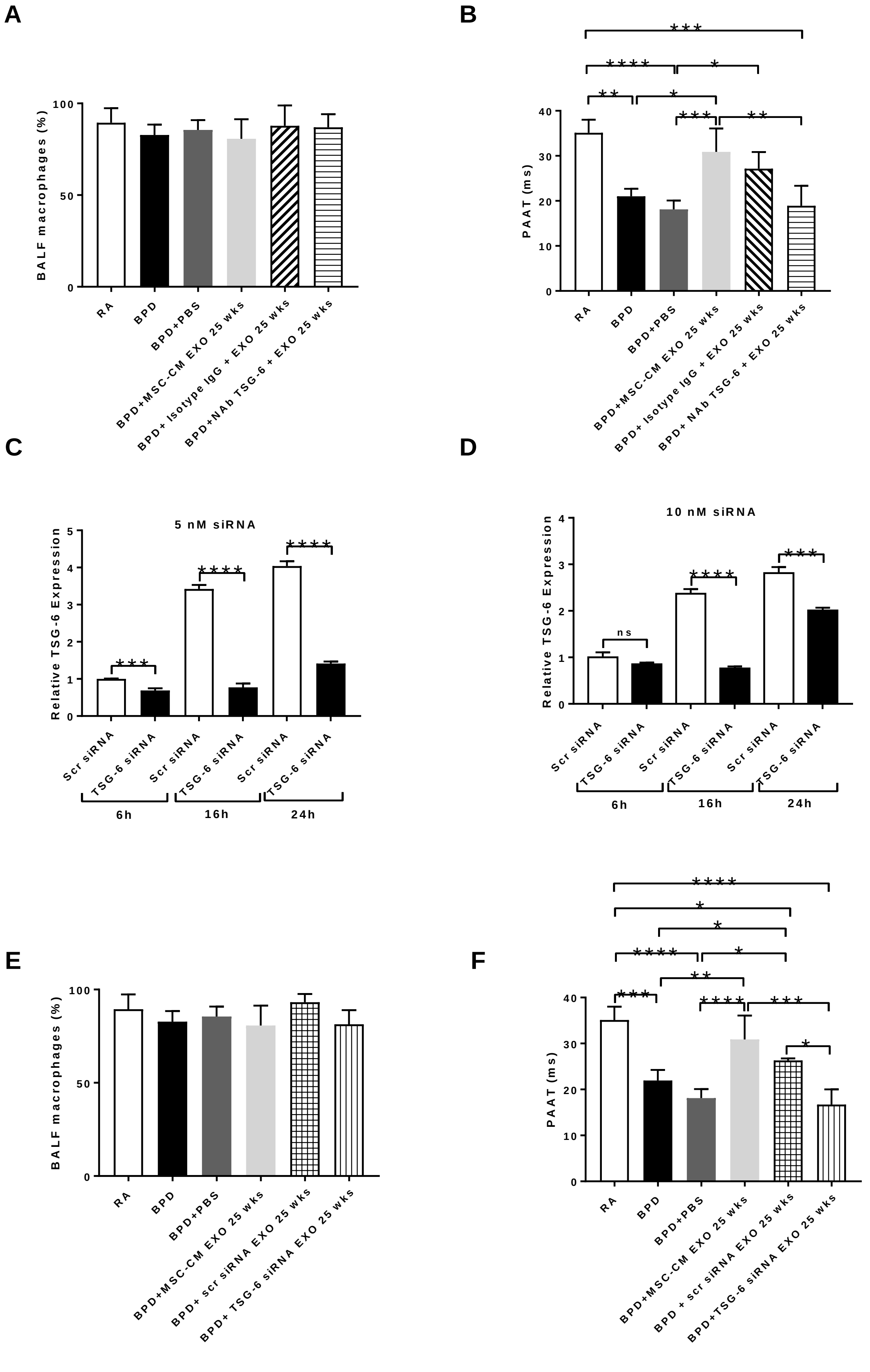

Supplement: Supplementary file 1 — Supplementary experimental procedures. Table S1. Comparison of different parameters in the exosome dose. Table S2. Clinical data for BPD and No BPD (n = 10). Figure S1. Preterm hUCs MSCs, but not fibroblasts, improve the BPD pulmonary phenotype. i.p. injections of MSCs from preterm (32 weeks) hUCs, in our mouse BPD model, at PN2 and PN4 showed improvement in lung architecture (at PN14), as evidenced by decreased chord length compared to the BPD mice. Human primary dermal fibroblast (HDF) cells, injected as control cells in the BPD mice, demonstrated no difference as compared to BPD mice. All values are expressed as mean ± standard deviation (SD); four experiments, N = 3 mice per group; one-way ANOVA with Tukey’s post hoc correction; *p < 0.05; **p < 0.01; ***p < 0.001. Figure S2. Isolation and characterization of preterm UC-MSCs (A) UC-MSCs from 25 and 30 wks gestational age UC, after Passage 1, 2 days (I, III) and 5 days (II, IV) in culture respectively. The cells were adherent to the culture flasks and displayed fibroblast-like morphology. (B) FACS characterization of UC-MSCs. UC-MSCs were positive for CD105, CD73 and CD90 and were negative for HLA-DR, CD19 and CD14. (C) Differentiation of UC-MSCs into adipocytes (Oil Red O staining), osteocytes (Alizarin Red S staining) and chondrocytes (Alcian Blue staining). Figure S3. (A-F). Isolation and identification of hUC MSC-CM EXO (A) Transmission electron micrograph (TEM) of hUC-MSC-EXO 25 wks (I) and 30 wks (II) depicts intact exosomes with a diameter of 40–120 nm. Scale bar: 100 nm. (B) Particle number and size of isolated exosomes by nanosight nanoparticle tracking analysis. Representative plot of the 25wks EXO and 30 wks EXO, samples 1:50 and 1:20 diluted, respectively, for the analysis. (C) Dot blot of UC-MSC-CM 25 wks (I), UC-MSC-CM 25 wks EXO (II), UC-MSC-CM 30 wks (III), UC-MSC-CM EXO 30 wks (IV), show positive signal for exosome-specific antibody CD63 while no signal is detected in negative controls - DMEM:F12 [file 13287_2018_903_MOESM1_ESM.pdf]
